# Evaluating and Enhancing an Educational Intervention to Reduce Smallholder Farmers’ Exposure to Pesticides in Uganda Through a Digital, Systematic Approach to Behavior Change: Protocol for a Cluster-Randomized Controlled Trial

**DOI:** 10.2196/55238

**Published:** 2024-05-08

**Authors:** Peter Ssekkadde, Vica Marie Jelena Tomberge, Curdin Brugger, Aggrey Atuhaire, Mohamed Aqiel Dalvie, Hanna-Andrea Rother, Martin Röösli, Jennifer Inauen, Samuel Fuhrimann

**Affiliations:** 1 Swiss Tropical and Public Health Institute Allschwil Switzerland; 2 University of Basel Basel Switzerland; 3 Institute of Psychology University of Bern Bern Switzerland; 4 Uganda National Association of Community and Occupational Health Kampala Uganda; 5 School of Public Health and Family Medicine University of Cape Town Cape Town South Africa

**Keywords:** smallholder farmers, knowledge, attitude, practice, behavior change, psychosocial determinants of behavior, health, education, pesticide exposure, SMS text messages

## Abstract

**Background:**

Smallholder farmers receive educational interventions on safe pesticide handling by governmental agencies, industries, or nongovernmental organizations to reduce exposure risks. However, existing educational interventions have limited effects on changing behaviors. Targeting psychosocial determinants of behavior change in educational interventions through theory- and evidence-based approaches may enhance their effectiveness.

**Objective:**

We aim at describing the intervention development and study design of a 3-arm cluster-randomized controlled trial to assess the effects in improving safe pesticide handling and reducing pesticide exposure of (1) an existing educational intervention and (2) a newly developed SMS text messaging intervention based on the Risks, Attitudes, Norms, Abilities, and Self-regulation (RANAS) behavior change approach.

**Methods:**

We enrolled 539 Ugandan smallholder farmers in 12 clusters (subcounties). The clusters, each with 45 farmers, were randomly allocated to one of the three arms: (1) educational intervention, (2) educational intervention+RANAS-based SMS text messages, or (3) control group. The educational intervention comprised a 2-day workshop that targeted multiple aspects of safe pesticide handling, whereas the SMS text messages targeted the use of personal protective equipment (PPE) and were based on the RANAS approach. For intervention development in this study, this approach includes identifying psychosocial determinants of PPE use at baseline and selecting behavior change techniques to target them in SMS text messages. The primary outcomes of the study are (1) pesticide knowledge, attitude, and practice scores indicating performance throughout the educational intervention; and (2) frequency of PPE use. Secondary outcomes are the RANAS-based behavioral determinants of PPE use, the frequency of glove use, algorithm-based pesticide exposure intensity scores, and signs and symptoms of pesticide poisoning. The outcomes were assessed in structured interviews before the intervention (baseline) and at the 12-month follow-up. The effect of the interventions among the arms will be analyzed using the intervention arms and baseline measures as predictors and the follow-up measures as outcomes in linear multivariable mixed models including the clusters as random effects. The mediating psychosocial determinants of the interventions will be assessed in multiple mediation models.

**Results:**

The study was conducted from 2020 to 2021—baseline interviews were conducted in October 2020, and the educational intervention was delivered in November 2020. The RANAS-based SMS text messages were developed based on the baseline data for relevant behavioral determinants of PPE use and sent between February 2021 and September 2021. Follow-up interviews were conducted in October 2021. Overall, 539 farmers were enrolled in the study at baseline; 8.3% (45/539) were lost to follow-up by the end of the study.

**Conclusions:**

This study will contribute to a better understanding of the effectiveness and behavior change mechanisms of educational interventions by using an experimental, cluster-randomized study design to improve pesticide handling among smallholder farmers.

**Trial Registration:**

International Standard Randomised Controlled Trial Number (ISRCTN) 18237656; https://doi.org/10.1186/ISRCTN18237656

**International Registered Report Identifier (IRRID):**

DERR1-10.2196/55238

## Introduction

### Background

Agricultural pesticide use has almost doubled worldwide in the past 3 decades, with a particular increase in low- and middle-income countries (LMICs) [1]. For example, in Uganda, there was a 12-fold increase in pesticide import value between 2000 (US $9.4 million) and 2020 (US $108.5 million) [2]. The increase in pesticide use results from many factors, such as the intensification of agricultural systems, changing land use patterns due to climate change [3], pesticide resistance, or increasing pest pressure [4]. In most LMICs, smallholder farmers dominate agricultural production [5], mainly relying on pesticides as their primary and often their only pest control strategy [6,7]. However, smallholder farmers often have insufficient knowledge, attitude, and practice (KAP) along the pesticide-handling chain, for example, limited knowledge of hazard information present on pesticide labels [8], low risk perception of the product [6,9], and limited use of recommended personal protective equipment (PPE) [6]. Hence, these gaps in the KAP of pesticide handling may risk farmers’ health [10,11].

The agricultural extension service system that ought to provide technical advice to improve farmers’ skills in pest control [12] and on safe pesticide handling [13] is often constrained by low staffing in LMICs. Therefore, it does not effectively reach out to farmers [4]. Consequently, smallholder farmers obtain most of their pesticide use information from their counterparts and agro-dealers [6]. However, agro-dealers often lack sufficient legal and technical qualifications to guide farmers on safe pesticide handling in LMICs [10,14]. Alongside these information channels, interventions promoting safe pesticide handling by smallholder farmers are rolled out by governmental agencies, industries, or nongovernmental organizations (NGOs) worldwide [15]. These interventions can be classified as educational or behavioral [16], technological or engineering-based, legislation- or enforcement-focused, incentive-based, and multifaceted interventions [15]. They range from creating awareness to promoting the use of PPE, adherence to manufacturers’ instructions, use of low-toxicity–class pesticides and other pest control strategies, and policy advocacy. By 2022, a total of 3 intervention studies had been systematically assessed for their impacts in reducing pesticide exposure in Africa [17]. Of these studies, 2 provided insight into the importance of farmer field schools in promoting integrated pest management (IPM) and, consequently, reducing pesticide use among smallholder farmers [18,19]. The other study provided evidence that training health care workers improves their knowledge and skill in handling acute pesticide poisoning cases [20].

In Uganda, educational interventions on pest management and safe pesticide use are mainly conducted by NGOs or industries [21]. These educational interventions, for example, reduced pesticide application frequency, increased PPE use, and introduced IPM strategies [18]. Similar findings are reported in other LMICs. For example, in Bolivia, trained farmers that adhered to safe pesticide use practices had considerably lower urinary pesticide metabolite levels after pesticide application than the untrained farmers [22]. Similarly, in Costa Rica, lower self-reported pesticide exposure intensity scores (EISs) among farmers were observed in farmers who had attended training in safe pesticide handling [23]. In summary, the existing literature on pesticide intervention evaluations in LMICs is limited by (1) cross-sectional study designs; (2) no comparative assessment of the impact on KAP; and (3) no systematic approach to targeting and assessing behavioral determinants of pesticide handling, such as farmers’ psychosocial cognitions [24].

One way to systematically change behavior is the Risks, Attitudes, Norms, Abilities, and Self-regulation (RANAS) framework [25]. The RANAS approach is an established tool that suggests different steps for designing and evaluating evidence- and theory-based behavior change interventions based on proposed psychosocial behavioral determinants [26]. This study protocol showcases the study design of a randomized controlled trial to evaluate and an enhance existing educational interventions using the RANAS approach for systematic behavior change [25] to promote safe pesticide handling among smallholder farmers in Uganda. This study is part of the African Pesticide Intervention Project, which seeks to promote and improve intervention studies on pesticide handling in LMICs [27].

### Aims and Specific Objectives of the Study

**Figure 1:**
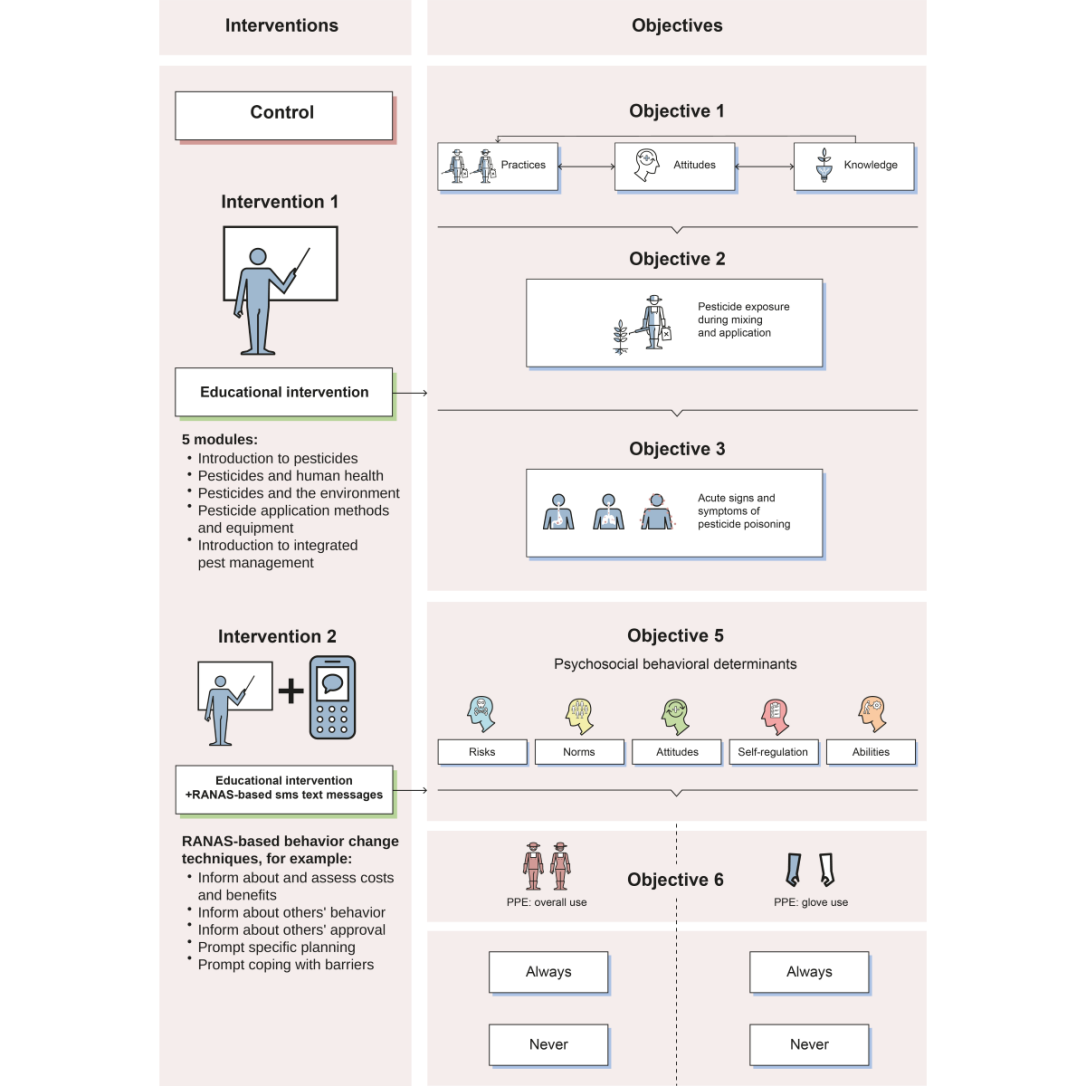
Conceptual framework showcasing the 5 objectives and their outcome measures to evaluate the existing educational intervention (2-day workshop) and the newly designed Risks, Attitudes, Norms, Abilities, and Self-regulation (RANAS)–based SMS text messaging intervention on safe pesticide handling among smallholder farmers in Uganda. PPE: personal protective equipment.

There are two main aims to this study (Figure 1): o evaluate the effectiveness of an existing educational intervention on pesticide-related KAP, PPE use, exposure, and health risks of pesticide handling; and (2) to evaluate whether the effect of the educational intervention can be enhanced by sending tailored RANAS-based SMS text messages targeting behavioral determinants of PPE use among smallholder farmers in Uganda. In relation to the first aim, the following outcomes are addressed:

1. To investigate the effect of the educational intervention on pesticide-related KAP along the educational curriculum compared to the control.
2. To assess the effect of the educational intervention on pesticide exposure intensity compared to controls.
3. To assess the effect of the educational intervention on health signs and symptoms associated with acute pesticide poisoning compared to controls.

In relation to aim 2, the specific research questions are as follows:

1. To test whether SMS text messages that targeted behavioral determinants based on the RANAS approach for behavior change have an additional, increased effect on PPE use compared to the educational intervention alone.
2. To understand which behavioral determinants specified by the RANAS approach were enhanced by the intervention and explain behavior change regarding PPE use.

## Methods

### Study Design

**Figure 2:**
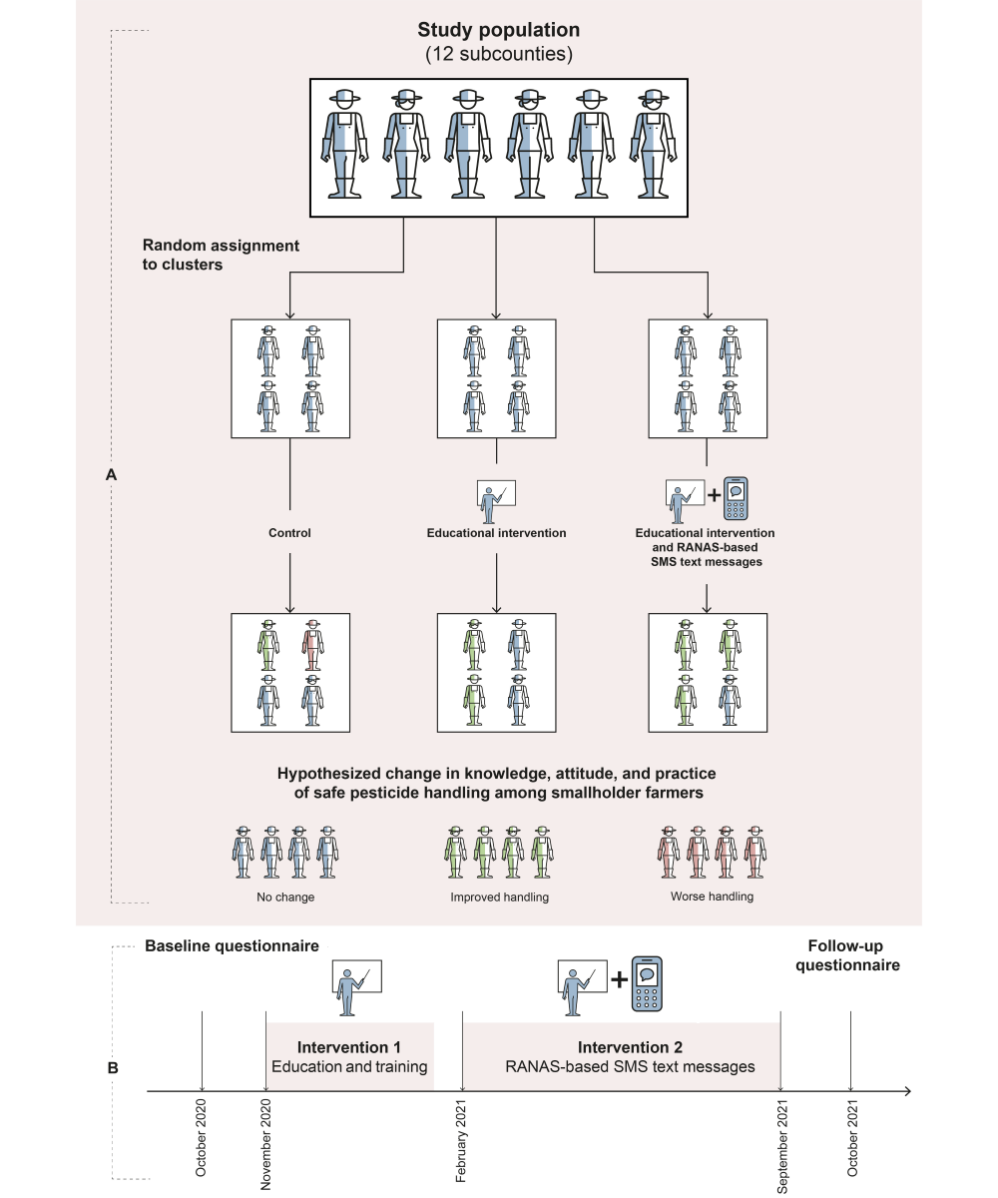
(A) Study design of the 3-arm cluster-randomized controlled trial to evaluate the educational intervention (2-day workshop) and the newly designed Risks, Attitudes, Norms, Abilities, and Self-regulation (RANAS)–based SMS text messaging intervention on safe pesticide handling. (B) Timeline of the 3-arm cluster-randomized controlled trial.

A cluster-randomized controlled trial with 3 arms—educational intervention, educational intervention and RANAS-based SMS text messages, and a control arm (received no intervention; Figure 2)—was conducted in the Kumi and Sembabule districts of Uganda between October 2020 and October 2021. A video describing the study can be found in Multimedia Appendix 1. A total of 12 subcounties (6 from either district), which are geographical administrative units of lower local government, formed the clusters for the study.

### Study Area

**Figure 3:**
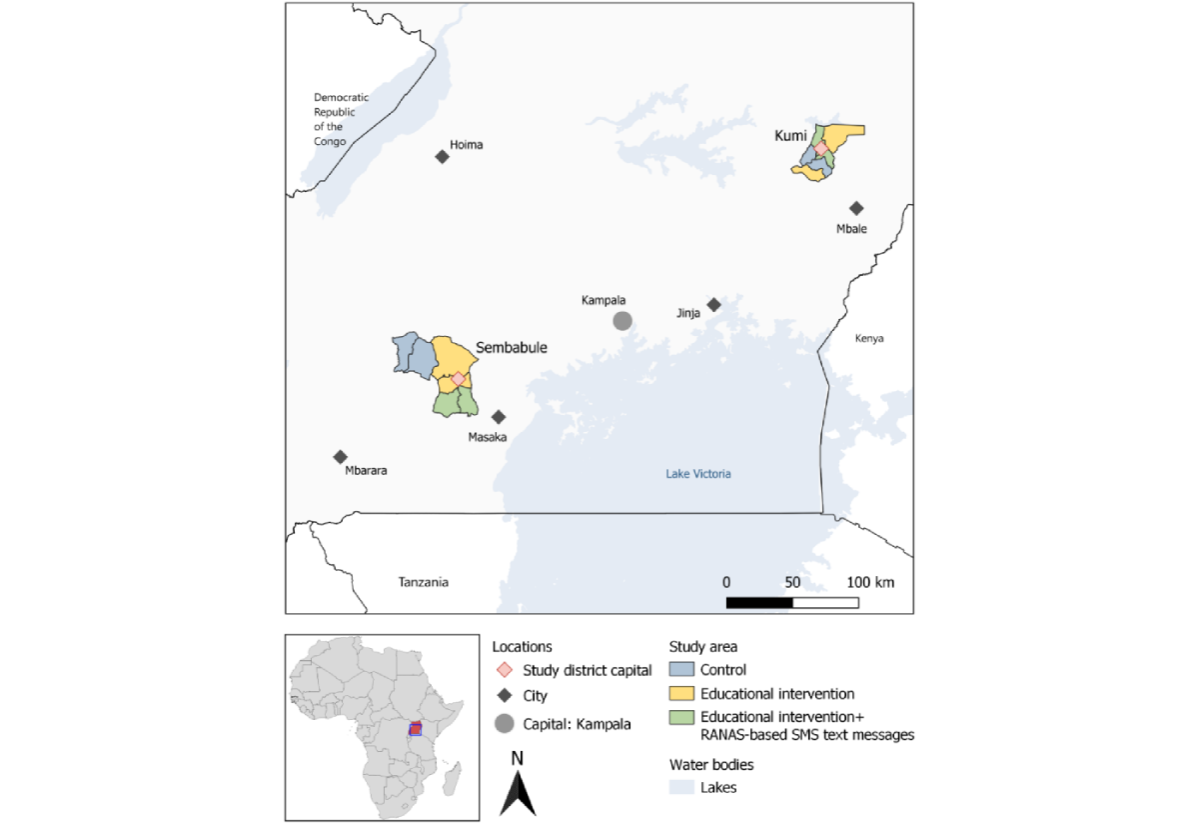
A map of Uganda showing the 2 study districts and intervention allocation of the subcounties. RANAS: Risks, Attitudes, Norms, Abilities, and Self-regulation.

Sembabule District is located in the central region of Uganda and has 6 subcounties and 2 town councils. Kumi District, on the other hand, is situated in the eastern region and subdivided into 6 subcounties and 1 municipality (Figure 3). The 6 subcounties from each district were selected based on their rural setting associated with ongoing agricultural activities. Both districts have pesticide-intensive crop (eg, watermelon, tomato, cabbage, and passion fruit) and livestock (eg, cattle, goats, and sheep) production systems, which constitute major economic activities.

### Participants

The District Farmers’ Associations (DFAs)—the Sembabule DFA and the Kumi DFA—provided lists of farmers who (1) grew at least one of the crops (watermelon, tomato, cabbage, and passion fruit) that are locally associated with frequent pesticide use, (2) had been actively involved in pesticide application in the previous 12 months, (3) were aged ≥18 years, (4) were able to read and write in English or the local language, (5) belonged to a household with at least one active mobile phone, and (6) had not directly participated in previous Uganda National Association of Community and Occupational Health (UNACOH)–led trainings in the area. Each of the DFAs provided a list each comprising 300 smallholder farmer names and contacts from their database, providing a total of 600 eligible participants, 50 from each subcounty. The research assistants contacted the farmers and interviewed (at baseline and 12 months afterward) those available and obtained consent until 45 farmers per subcounty were enrolled. The interviews took place at the farmers’ homes or fields depending on the farmers’ location at that time.

### Ethical Considerations

Ethics approval for the study was secured from the Higher Degrees, Research, and Ethics Committee at Makerere University in Uganda (reference 846). In addition, the study was registered on the ISRCTN clinical trial registry (ISRCTN 18237656), and written informed consent was obtained from all study participants before the baseline interview.

### Randomization and Blinding

After the baseline survey in October 2020, a total of 4 clusters each with 45 farmers were randomly assigned to one of the three arms, comprising 180 farmers per arm (Figure 1): (1) educational intervention, (2) educational intervention+RANAS-based SMS text messages, or (3) control group. One of the Swiss investigators who had no previous knowledge of the subcounties used Microsoft Excel (Microsoft Corp) random numbers to randomize the clusters to the arms. The allocation of the clusters was concealed from the participants. The participants were also blinded with regard to which arm other farmers had been assigned to. The investigators and facilitators knew which arm the farmers had been assigned to, and at the follow-up interviews, the research assistants could know which arm the farmers had been assigned to.

### Sample Size Calculation

We estimated a sample size of 540 farmers that assumed a statistical power of 80% based on the estimated observed differences between the EISs of an average pesticide application between smallholder farmers who received training in pesticide handling and those who did not receive it in a recent survey in Uganda [23]. The trained group had a mean EIS of 0.47 (SD 0.10), and the untrained group had a mean EIS of 0.55 (SD 0.18). We assumed that the 2 planned intervention arms would show a similar effect distribution. Hence, we based our sample size calculation on the 2-sample means test assuming a cluster-randomized design, a total of 12 clusters equally distributed between the control and intervention groups, and an intraclass correlation coefficient of 0.4. The package *power two means* in the statistical software Stata (version 15; StataCorp) resulted in a cluster size of 40 farmers and a total of 480 farmers, which would reach a power of 80% at a significance level of 5%. To account for a possible dropout rate of 13% at follow-up based on a previous study in a similar setting [23], in each cluster, an additional 5 farmers were enrolled, hence constituting a total sample size of 540.

### Description of the Interventions

#### Educational Intervention

The investigated educational intervention is an existing training curriculum (Multimedia Appendix 2) designed by the UNACOH to reduce pesticide exposure and associated human and environmental health effects in Uganda between 2010 and 2020 [28]. It includes 5 modules: introduction to synthetic pesticides, pesticides and human health, pesticides and the environment, pesticide application equipment, and IPM. The main objective of the intervention was to improve pesticide handling through enhancing the KAP of smallholder farmers. Furthermore, the intervention is hypothesized to reduce pesticide exposure and the negative associated health symptoms.

The intervention was developed under the Pesticide Use Health and Environment (PHE) project and implemented in 3 phases by the UNACOH in collaboration with Diálogos, a Danish NGO. The project’s interventions included 3 thematic areas: evidence gathering or documentation, training or knowledge dissemination, and policy influence or lobbying and advocacy. Among the several project trainees were smallholder farmers, agro-dealers, extension workers, village health personnel, and health workers on topics such as responsible pesticide handling; IPM; and acute pesticide poisoning diagnosis, management, and reporting.

The educational materials used by the PHE project in its first phase (2010-2013) were originally adapted from a sister project, *Proyecto De Desarrollo Comunitario*, implemented in Bolivia with support from the same partner, “Dialogos.” A series of 7 small booklets or modular training guides was developed and used to facilitate training in the first 2 phases (2010-2013 and 2013-2017), and at the start of the third phase (2017-2020), they were revised and merged into 1 training manual or curriculum in technical partnership with and with approval from the Agricultural Chemicals Control Board; Department of Crop Protection; and Ministry of Agriculture, Animal Industry, and Fisheries of Uganda. Copies of this 5-module manual, titled “Responsible pesticide use and handling: A guide for sustainable pest management,” were widely disseminated by the Ministry of Agriculture, Animal Industry, and Fisheries through its structures.

#### RANAS-Based Text Messages

##### Overview

**Figure 4:**
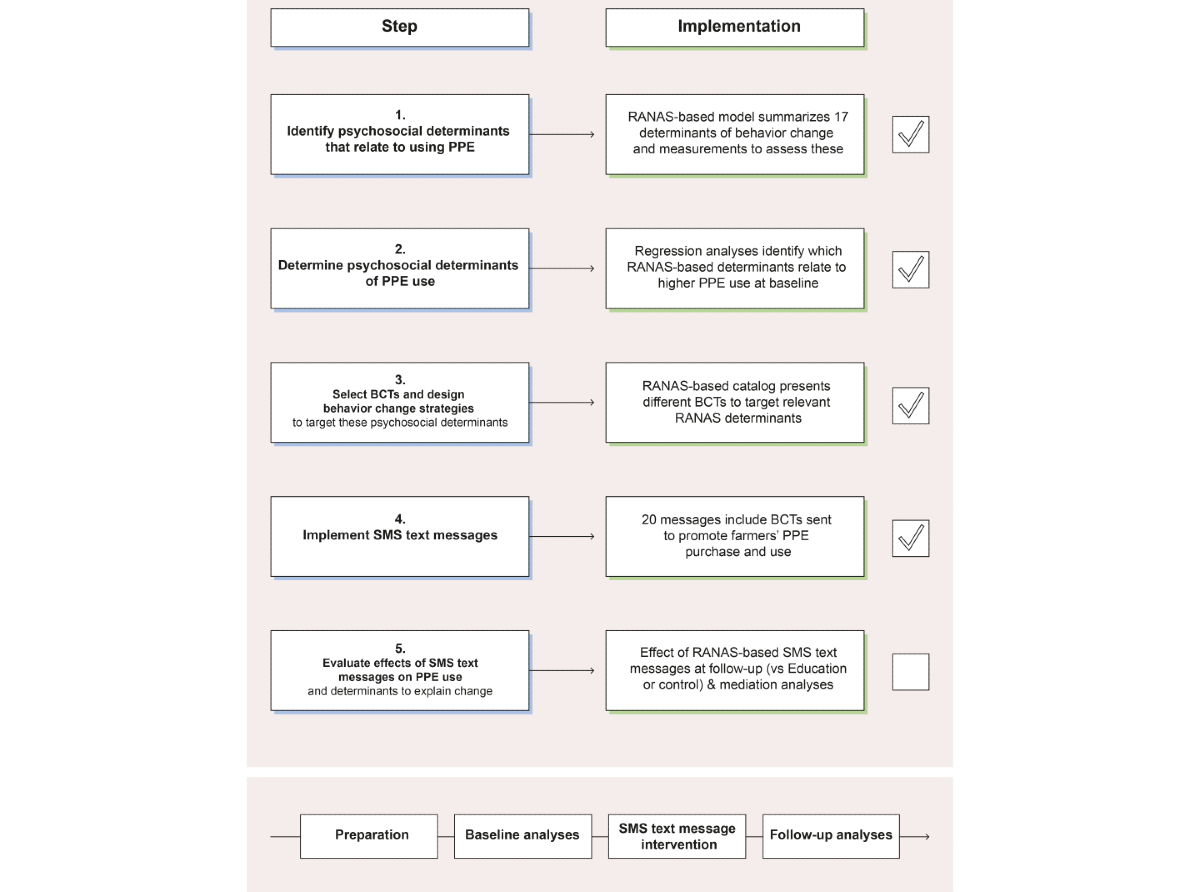
Development and evaluation guide for the SMS text messaging intervention based on the steps to systematic behavior change from the Risks, Attitudes, Norms, Abilities, and Self-regulation (RANAS) approach [25] for this study. BCT: behavior change technique; PPE: personal protective equipment.

In addition to being delivered the educational training manual, one arm received mobile phone SMS text message reminders. The SMS text message intervention was based on the RANAS approach, a systematic approach for behavior change (see Multimedia Appendix 3 for detailed wordings of the messages). The RANAS approach is an established tool that suggests different steps for designing and evaluating evidence- and theory-based behavior change interventions, most importantly by informing intervention development through a baseline assessment (see step 1). Figure 4 outlines the 5 steps suggested by the RANAS approach and how these steps were applied to design the SMS text messages in this study. All steps have been successfully executed (see the ticked boxes in Figure 4) except for the evaluation phase, which will be reported in forthcoming papers. Thus, we promoted farmers’ PPE use as one behavior to substantially reduce pesticide exposure [23].

##### Step 1 of RANAS-Based Intervention Development: Identifying Psychosocial Determinants of PPE Use at Baseline

The core concept of the RANAS approach is that behavior change is driven by various psychosocial determinants that need to be in favor of new behavior [26,29]. On the basis of a synthesis of behavior change theories (eg, the health belief model [30] and the theory of planned behavior [31]), the RANAS approach describes 17 psychosocial determinants that are of potential relevance to change behavior. These are grouped into 5 categories of determinants (risks, attitudes, norms, abilities, and self-regulation). Table 1 outlines sample items for all determinants proposed by the RANAS approach, which can help understand their definition.

1. Risks comprise health knowledge, perceived vulnerability (estimates regarding the personal likelihood of catching a disease related to pesticides), and severity (estimates regarding the seriousness of a pesticide-related disease and its consequences).
2. Attitudes include feelings (emotions arising when thinking of or practicing PPE use or thinking about its consequences, eg, being proud of using PPE) as well as beliefs about cost benefits, such as monetary and nonmonetary (eg, farmers’ perception of the fit of PPE and expenditure of time related to PPE use) costs and benefits of a behavior. These beliefs may also include response efficacy, which is the belief of successfully averting threats by using PPE.
3. Norms include perception of descriptive norm (others’ behavior, ie, observation and awareness of PPE use practiced by others) and injunctive norm (perception of to what extent PPE use is typically approved or disapproved of by significant others and institutional norms), as well as personal importance (farmers’ belief of what the farmer should [not] do).
4. Abilities include how-to knowledge (knowledge of how to obtain access to PPE) and confidence (perceived ability to execute and continue PPE use and recover from setbacks of not using PPE).
5. Self-regulation includes action planning (attempts to plan for PPE use, eg, when, where, and how to buy or wear PPE), action control (attempts to self-monitor PPE use and correcting it toward a behavioral goal), barrier planning (attempts to plan to overcome barriers that would impede PPE use), commitment (the obligation that a farmer feels), and effort to practice PPE use.

**Table 1:** Baseline results to identify relevant psychosocial determinants of personal protective equipment (PPE) use based on the Risks, Attitudes, Norms, Abilities, and Self-regulation approach—(1) intention to buy PPE, (2) intention to use PPE, (3) amount of PPE items owned, and (4) frequency of PPE use.

Predictor	Sample item	Amount of PPE items owned, β (SE)	Frequency of PPE use, β (SE)	Intention to buy PPE, β (SE)	Intention to use PPE, β (SE)
Intercept	N/A^a^	.47 (0.09)^b^	.27 (.08)^b^	.06 (.11)^b^	.34 (.07)^b^
Intention to buy PPE	How strong is your intention to buy all the recommended PPE items for your personal use?	.05 (.04)	.04 (.03)	N/A	N/A
Intention to use PPE	How strong is your intention to always wear all the recommended PPE items when handling pesticides in the future?	–.01 (.05)	–.04 (.05)	—	—
Vulnerability	How likely do you think it is that you will experience acute symptoms from using pesticides if you do not wear all the recommended PPE items?	–.00 (.05)	.01 (.04)	.15 (.06)^c^	.01 (.04)
Severity	Imagine you are experiencing acute symptoms of pesticide use, how severe do you rate the consequences for your own health?	.02 (.05)	–.03 (.05)	–.17 (.07)^c^	–.06 (.05)
Response efficacy	To what extent do you think that wearing all the recommended PPE items when handling pesticides can protect you from getting sick?	–.04 (.05)	.01 (.04)	.11 (.06)	.20 (.04)^b^
Attitude: costs	How expensive do you consider it to buy all the recommended PPE items?	–.09 (.04)^c^	–.08 (.04)^c^	–.01 (.05)	.02 (.03)
Attitude: fitting clothes	How fitting is the recommended PPE for you (eg, size and cut)?	.05 (.04)	.03 (.04)	.04 (.05)	–.01 (.04)
Attitude: time consuming	How time consuming do you think it is to put on all the recommended PPE items when handling pesticides?	.02 (.03)	.01 (.03)	.12 (.04)^d^	–.00 (.03)
Feeling: uncomfortable	To what extent do you feel that it is uncomfortable to always wear all the recommended PPE items when handling pesticides? (recoded)	.04 (.03)	.04 (.03)	.06 (.04)	–.01 (.03)
Feeling: proud	How proud would you feel if you wore all the recommended PPE items when handling pesticides?	.03 (.05)	.04 (.04)	.17 (.06)^d^	–.01 (.04)
Feeling: weak	To what extent would others think you are weak when wearing all the recommended PPE items? (recoded)	–.04 (.02)	–.03 (.02)	–.02 (.03)	.02 (.02)
Overall rate of benefit	Now, considering the potential disadvantages of wearing all the recommended PPE items against its advantages, how advantageous do you evaluate the use of PPE as recommended?	–.03 (.06)	.04 (.06)	.00 (.08)	.08 (.05)
Descriptive norm (others’ behavior)	How many farmers in neighboring farms are using all the recommended PPE items when handling pesticides?	.40 (.06)^b^	.36 (.05)^b^	–0.12 (.07)	.04 (.05)
Injunctive norm (others’ approval)	To what extent do you think that your wife (or partner) wants you to wear all the recommended PPE items when handling pesticides?	–.01 (.04)	.00 (.04)	.13 (.05)^c^	.01 (.04)
Personal norm	How important is it for you to wear all the recommended PPE items when handling pesticides?	–.01 (.07)	.02 (.07)	.15 (.09)	.07 (.06)
Action knowledge	Where would you go to buy all this equipment?	.05 (.02)^d^	.04 (.02)^c^	.01 (.02)	–.00 (.02)
Confidence	How confident are you that you can remember to wear all the recommended PPE items when handling pesticides?	.00 (.04)	–.01 (.04)	.08 (.06)	.04 (.04)
Action plan to buy PPE	Do you have a plan for how to buy all the PPE items to cover all body parts?	.06 (.05)	.03 (.04)	.23 (.06)^b^	.01 (.04)
Action plan to wear PPE	Imagine you have all the PPE items, do you have a plan to make sure you always wear them?	.05 (.04)	.10 (.04)^c^	.09 (.05)	.04 (.04)
Barrier planning	Do you have a plan for how to overcome these difficulties (that hinder you from wearing PPE)?	–.31 (.06)^b^	–.28 (.05)^b^	–.06 (.07)	–.01 (.05)
Commitment	How strongly do you feel committed to wearing the PPE items you have when handling pesticides?	.02 (.05)	.08 (.04)	–.04 (.06)	.06 (.04)
Effort	To what extent do you make a conscious effort to always wear all the recommended PPE items?	–.00 (.03)	.01 (.03)	.09 (.04)^c^	.18 (.03)^b^

^a^N/A: not applicable.

^b^*P*<.001.

^c^*P*<.05.

^d^*P*<.01.

##### Step 2 of RANAS-Based Intervention Development: Identifying Behavioral Determinants of PPE-Related Intentions and Behaviors at Baseline

Different contexts, behaviors, and persons may presuppose the expression of different psychosocial behavioral determinants, which need to be identified in a baseline assessment (step 2). To identify the determinants that are relevant when promoting PPE use among Ugandan smallholder farmers, we assessed all psychosocial determinants described by the RANAS approach at baseline (see the Outcome Measures section; ie, psychosocial determinants related to pursuing PPE use and using PPE). Multiple regression analyses were then computed to identify which determinants were related to higher behavior and intention to pursue PPE use and use PPE. In total, 4 regression models were calculated using the following dependent variables: intention to buy PPE, intention to use PPE, number of PPE items owned, and frequency of PPE use.

The baseline analyses of all 3 study groups before the interventions revealed that farmers owned PPE to cover an average of 4 (out of 8; SD 1.6) body parts. The accumulated frequency of using PPE to cover all body parts was 4.1 (SD 2.8), with a possible range of 0 (never covering any body part) to 40 (always covering all body parts). This mean corresponds to 10% (SD 7%) of full body, consistent use (always covering all body parts with PPE=100%). The regression analyses (Table 1) indicated the following:

1. Farmers’ intention to buy PPE was higher when they considered themselves more vulnerable to health risks, perceived the health risks of pesticides to be less severe, perceived wearing PPE as more time consuming, felt proud of using PPE, perceived other people to approve of PPE use more, and had an action plan for where to buy PPE, as well as when they were more committed to using PPE.
2. Farmers’ intention to buy PPE was higher when they perceived higher response efficacy and were more committed to using PPE.
3. Farmers owned more PPE items when they perceived the costs to be lower, perceived other farmers to use PPE, had an action plan for where to buy PPE, and had a coping plan in the sense of overcoming arising barriers to using PEE and pursuing more PPE.
4. Farmers used PPE more frequently when they perceived the costs to be lower, perceived other farmers to use PPE, had an action plan for where to buy PPE, and had a coping plan in the sense of overcoming arising barriers to using PPE pursued more PPE.

##### Step 3 of RANAS-Based Intervention Development: Selecting Behavior Change Techniques and Designing Behavior Change Strategies

After identifying relevant psychosocial determinants of the healthy behavior (in this case, PPE use) at baseline, the RANAS approach suggests selecting behavior change techniques to promote the identified psychosocial determinants. In this way, farmers who are not yet adopting the behavior may be enabled to do so. Behavior change techniques are components of an intervention designed to enable behavior change by targeting psychosocial determinants that facilitate behavior change [32,33]. The RANAS catalog proposes which behavior change techniques to select to target each RANAS-based psychosocial determinant. The behavior change techniques that were used, as well as detailed wording of the developed SMS text messages, can be found in Multimedia Appendix 3.

#### Procedures of Educational Intervention

A 2-day workshop was held in the DFAs’ training facilities and delivered the curriculum based on the 5 modules (see Multimedia Appendix 4 for a summary of activities per module as well as coded behavior change techniques). The training was facilitated by the study coordinator, the author of the educational curriculum, who has >10 years of field experience facilitating similar trainings under the PHE project. In total, 2 local extension workers supported the coordinator. Delivery methods for the educational intervention included short videos, practical demonstrations, visual aids (pesticide labels, PPE, and spraying equipment), farmer experience sharing, breakout group exercises, and take-home infographic posters.

#### Step 4 of RANAS-Based Intervention Development: Procedures of RANAS-Based Text Messages

**Figure 5:**
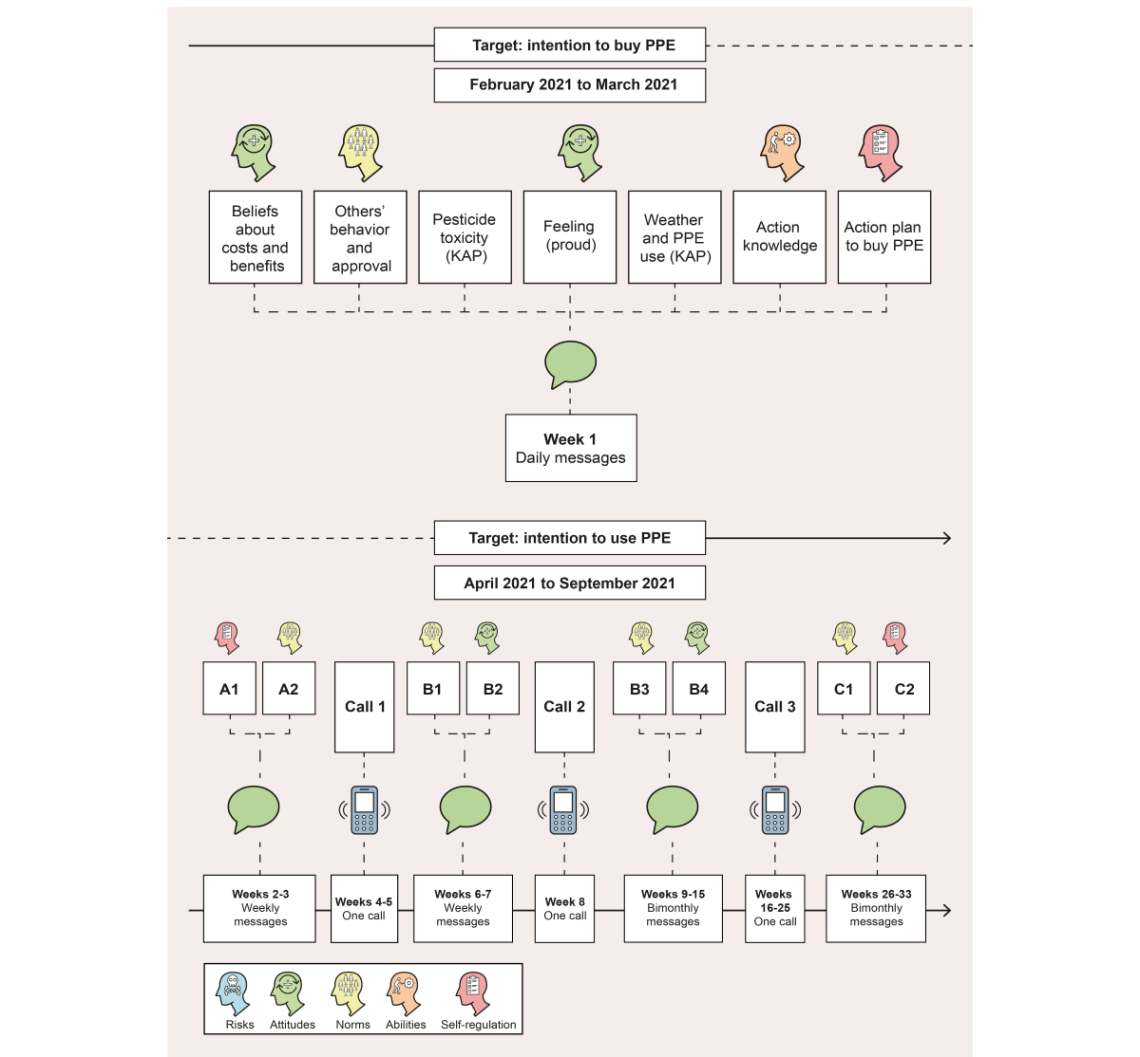
Procedures for the Risks, Attitudes, Norms, Abilities, and Self-regulation (RANAS)–based SMS text messaging intervention for smallholder farmers in Uganda. KAP: knowledge, attitude, and practice; PPE: personal protective equipment.

After the educational intervention was implemented, 20 SMS text messages were sent in increasing intervals from mid-February 2021 to September 2021 to the farmers assigned to 1 of the 2 arms that received the educational intervention. Figure 5 illustrates the procedure, targeted psychosocial determinants of behavior, and behavior change techniques. The first phase was implemented from February 2021 to March 2021 and aimed at motivating farmers to buy PPE (A1: action plan to buy PPE; A2: others’ behavior; call 1: action plan+monitoring of PPE purchase; B1: others’ behavior; B2: response efficacy, as indicated in the figure). The second phase was carried out from April 2021 to September 2021 with an emphasis on the use of PPE while handling pesticides during the spraying season (calls 2 and 3: monitoring of PPE use; B3: others’ behavior; B4: response efficacy; C1: others’ behavior; C2: overcoming barriers). The SMS text messages were automatically sent to the farmers’ phones according to the following timeline. In the first intensive week, we sent daily SMS text messages to refresh the RANAS framework and 2 KAP-related details that had been identified as relevant in the baseline assessments. These targeted beliefs about costs and benefits, descriptive and injunctive norm, attitudes toward pesticide toxicity (KAP), feelings (proud, not weak), knowledge of the best weather in which to use PPE (KAP), action knowledge, and action planning. Gradually decreasing the frequency of reminders, we then sent weekly; biweekly; and, finally, monthly SMS text messages. These alternatively targeted descriptive norm (others’ behavior), response efficacy, and barrier planning regarding PPE use. As one of the most relevant determinants was descriptive norm we wished to send real information about other farmers’ behavior to the participants, particularly other farmers’ extent of owning and using PPE. For this purpose, the participants were called 3 times during the SMS text messaging period to assess to what extent they owned and used PPE to later on share this information in SMS text messages with other farmers.

### Outcome Measures

#### Overview

The coprimary outcomes of the study are (1) pesticide KAP scores indicating performance along the educational curriculum and (2) PPE use (to cover all body parts). Secondary outcomes are the psychosocial determinants based on the RANAS framework regarding PPE use, frequency of using gloves when handling pesticides, algorithm-based pesticide EISs, and frequency of health signs and symptoms associated with pesticide poisoning. All measures were self-reported. The full questionnaire, programmed to be used in Open Data Kit (ODK; Get ODK Inc), can be found in Multimedia Appendix 5. Items using Likert scales had 5 answer options and were augmented with a visual 5-dot scale to increase participants’ understanding, as has been shown to be helpful in other studies to support responding populations with low literacy [26]. The outcomes are summarized in Textbox 1.

**Summary of the objectives and outcomes of the study.**

**Objective 1: knowledge, attitude, and practice on pesticide handling**

- Knowledge mean scores
- Attitude mean scores
- Practice mean scores

**Objective 2: pesticide exposure**

- Personal protective equipment (PPE) use exposure scores
- Change scores
- Changing cloths (CHANGE) exposure scores
- Exposure intensity scores (EIS)
- Yearly pesticide application days
- Yearly pesticide exposure (EIS^Year^)

**Objective 3: health signs and symptoms associated with pesticide poisoning**

- Sum of health signs and symptoms
- Category of symptoms (low and high based on the baseline median)

**Objective 4: effect on PPE use**

- Mean frequency of using 8 PPE items to cover the whole body [23,34]
- Mean frequency of using gloves [23,34]

**Objective 4: determinants of behavior change regarding PPE use**

- Mean score of the following [29,35]:
  Intention to buy PPE
  Intention to use PPE
  Vulnerability
  Severity
  Response efficacy
  Attitude: costs
  Attitude: fitting clothes
  Attitude: time consuming
  Feeling: uncomfortable
  Feeling: proud
  Feeling: weak
  Overall rate of benefit
  Descriptive norm (others’ behavior)
  Injunctive norm (others’ approval)
  Personal norm
  Action knowledge
  Confidence
  Action plan to buy PPE
  Action plan to wear PPE
  Barrier planning
  Effort
  Commitment

#### KAP Assessment

##### Overview

KAP scores were assessed based on 16 items grouped around a similar training module separately for KAP (the full list of questions can be found in Multimedia Appendix 6). The KAP items were developed based on previous KAP assessments in the Pesticide Use in Tropical Settings survey [21] and another study [36] and adapted to assess KAP for 4 out of the 5 modules of the educational intervention curriculum on pesticide handling. The fifth module on IPM was not considered for the KAP scores as it was mainly related to pesticide product use and is captured in the exposure frequency under objective 2.

##### Example KAP Scoring

Knowledge questions were binary (eg, “The name of the plant protection product active ingredients can be found on the label of the product” [*true*, *false*, or *not sure*]). For every correct answer, 1 point was added to the knowledge score, resulting in final scores ranging between 0 and 16. The higher the score, the more knowledge the farmer had on safe pesticide handling. The “Not sure” responses were treated as “False.”

Attitude was measured using a 5-point Likert scale (eg, “It is important for me to know the active ingredients in a given plant protection product” [*agree not at all*=1; *agree a little*=2; *somewhat agree*=3; *rather agree*=4; *strongly agree*=5; *do not have an opinion*=coded as missing]).

Practice was also measured using a 5-point Likert scale (eg, “I look for the name of the active ingredients when buying or using a given pesticide product” [*never*, 0%=1; *rarely*, 25%=2; *sometimes*, 50%=3; *often*, 75%=4; *always*, 100%=5; *do not have an opinion*=coded as missing]).

#### Pesticide Exposure

The pesticide EIS (ranging from 0 to 13) during an average application day was based on a semiquantitative exposure algorithm specifically modified [23], applied, and validated for smallholder farmers who use handheld knapsack sprayers in LMICs as described in recent studies [37-39]. Concisely, EIS estimates are derived from a comprehensive evaluation involving 5 key factors. These factors are (1) mixing pesticides (MIX; score of 5); (2) the application of pesticides using a knapsack (APPLICATION; score of 8); (3) the type of PPE used, protecting different body parts, and accounting for differences in application frequency (PPE; score of 0.14-1); (4) the time taken to change clothing after pesticide handling (CHANGE; score of 0.7-1); and (5) the postapplication bathing routine (SHOWER; score of 0.7-1), as illustrated in equation 1. Mixing and application of pesticides are expected to increase exposure, whereas use of PPE, changing clothes, and showering after an application reduce exposure.

EIS = (MIX + APPLICATION) x PPE x CHANGE x SHOWER **(1)**

Yearly pesticide exposure was assessed using [23] (1) the yearly pesticide application days over the previous 12 months and (2) the EIS during an average application day, as indicated in equation 2. The pesticide application days were captured using a spreadsheet that included the pesticide products used by the farmer in the previous 12 months and the number of days each product was applied [39.

EIS^Year^ = EIS × Yearly pesticide application days **(2)**

#### Health Signs and Symptoms Associated With Acute Pesticide Poisoning

Farmers’ health was assessed using a checklist of 31 symptoms associated with acute poisoning (eg, dizziness, anxiousness, nausea, vomiting, sleeping difficulties, weakness of the limbs, and changes in taste and smell [40]). Participants were asked whether they presented the symptoms (yes or no) and, if they did, the frequency with which they experienced them (once a year, once a month, once a week, or more than once a week) and whether they experienced them after spraying pesticides. The sum of the symptoms experienced by the farmers after pesticide application will be calculated and further dichotomized based on the baseline median distribution of the signs and symptoms.

#### Frequency of PPE Use and its Psychosocial Determinants

Using PPE to cover all body parts was assessed using behavioral frequency measures as is common in behavioral science [34]. Participants were shown pictures of 15 PPE items to cover the whole body and were asked about the frequency of using these items in the previous 12 months, with answer options ranging from *always*=1 to *never*=5. These items were reverse coded as infrequent use (1) and frequent use (5) and categorized into 8 means for the covered body parts: “eyes, mouth, hands, arms, legs, feet, trunk, and head” [23]. To create the total frequency of PPE use to cover all body parts, a sum of all categories’ means was calculated (ie, frequency of PPE use = mean [frequency of using items to cover the hands] + mean [frequency of using items to cover the mouth] + ... + mean [frequency of using items to cover the feet).

Frequency of using gloves*.* In addition, as the hands are the most exposed body part during pesticide handling [23], we assessed the mean frequency of using PPE to cover the hands through common behavioral frequency assessment [34]. Participants were asked about the frequency of using PPE to cover their hands (using any kind of gloves).

Exposure modified by PPE use was assessed using the modifying PPE variable from the pesticide exposure scores [23]. This can account for the material of the PPE being used and the exposure risk of different body parts. For example, the hands account for 40% of pesticide exposure, whereas the eyes-only account for 10% of exposure.

Psychosocial determinants of PPE use (RANAS factors) were assessed using the measurements proposed by the RANAS model [29,35]. All items were pretested in an unpublished pilot study in Uganda. The items assessed the 17 psychosocial determinants proposed by the RANAS approach asking participants how much they agreed with certain RANAS-based questions (see Table 1 in the *Description of the Interventions* section for sample items) using a 5-point Likert scale (*not at all*=1; *very much*=5). For each scale, multiple items were averaged. Beliefs about cost benefits in the attitude category were assessed using single rather than averaged items given their low internal consistency at baseline.

Sociodemographic characteristics included age, educational level, marital status, number of children, smoking status, drug and alcohol use, main occupation, and monthly income. Farming characteristics included farm size, main farming objective, number of farm workers, major crops grown, main pest management practices, debut age for working on farms, age when they started mixing and applying pesticides, who mixes the pesticides, disposal of pesticide containers, who washes the pesticide application equipment, field re-entry after pesticide application, sources of farming and pesticide use information, training in pesticide use, knowledge of routes of exposure, and negative consequences of pesticide use.

### Data Collection and Management

The study data were collected using an electronic questionnaire developed using ODK. The research assistants were trained before the baseline interviews on the objectives of the study, ethical aspects, and the use of the ODK. They collected pretest data in a pilot study from 20 smallholder farmers from another district that was not part of our study. The research assistants visited the farmers either in their homes or on the fields and administered the computer-assisted interviews in either 3 local dialects (Luganda, Runyankore, or Ateso) or English. At the end of each day, the data were uploaded to a secure server. A data quality management team inspected the data regularly for any inconsistencies or errors, and where found, the responsible research assistants were contacted, and appropriate resolutions were reached. The data were securely stored in a password-protected database with restricted access.

### Statistical Analyses

Statistical analyses will be conducted using Stata (version 15; StataCorp) and R (version 4.3.1; The R Foundation). The outcomes will be based on intention-to-treat analyses, and missing data values will be computed using the multivariate imputation chained equations R package. The effect of the interventions on the 3 arms will be analyzed using the intervention arms as dummy-coded predictors and the follow-up measures as outcomes in multivariate mixed models with the cluster (subcounty) as the random effect. Sensitivity analyses are planned to control for differences in sex, age, educational level, previous training, and annual pesticide use days. For objective 2, subanalyses of factor scores (type of PPE, time taken to change clothing after pesticide handling, postapplication bathing routine, mixing pesticides, and application of pesticides using a knapsack) used to compute the exposure algorithms will be made to test their variation resulting from the interventions.

Intervention effects on KAP, PPE use (frequency of using PPE for all body parts, frequency of using gloves, and exposure modified by pesticide use), algorithm-based pesticide EIS, and health signs and symptoms associated with acute pesticide poisoning will be analyzed in a model testing the control group (0) against the educational intervention (1) and against the educational intervention+RANAS-based SMS text messages (1). For the intervention effects on PPE and glove use (objective 4), all arms will be compared by testing the educational intervention against the educational intervention+RANAS-based SMS text messages (0) in an additional model.

To understand the psychosocial mechanisms of the interventions (objective 5), we will perform 2 mediation models with multiple psychosocial behavioral determinants as mediators, one testing the educational intervention group (1) against the control group (0) and one testing the educational intervention+RANAS-based text message group (1) against the educational intervention (0). The mediation analyses will use the change scores (follow-up value – baseline value) for all RANAS-based psychosocial determinants as mediators and PPE use as the outcome, operationalized as the frequency of using PPE to cover all body parts and using gloves.

## Results

The study received approval from Makerere University’s Higher Degrees, Research, and Ethics Committee in Uganda (reference 846) and was additionally registered in the ISRCTN clinical trial registry (ISRCTN 18237656). It was conducted between October 2020 and October 2021, and 539 farmers in 12 clusters were enrolled. Data were collected from October 5 to 30, 2020, for the baseline assessment, and for the follow-up, data collection occurred from October 1 to 26, 2021.

**Figure 6:**
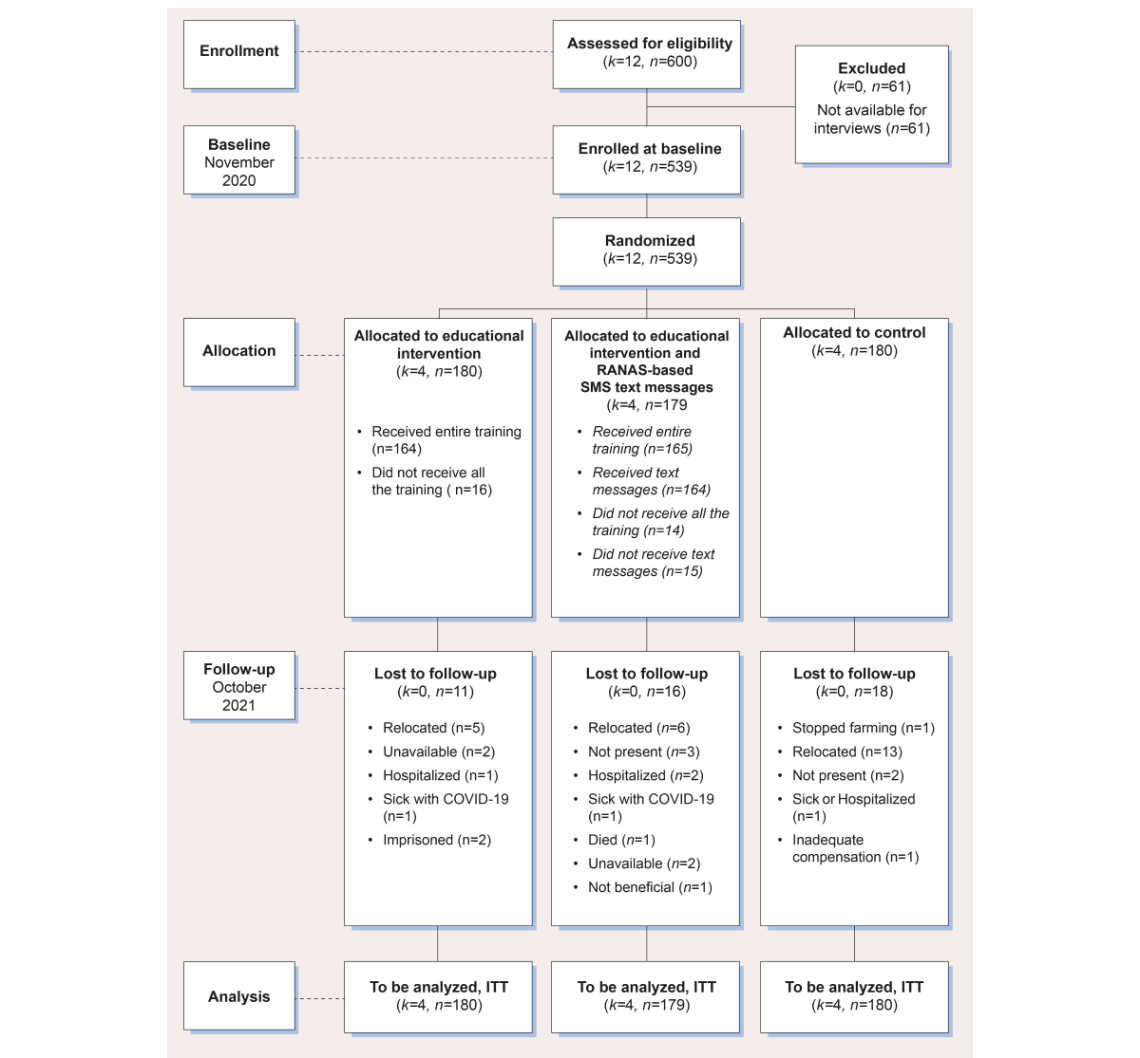
African Pesticide Intervention Project (APSENT) consent flowchart showing participant recruitment and inclusion and exclusion from the educational and SMS text messaging interventions. ITT: intention to treat; RANAS: Risks, Attitudes, Norms, Abilities, and Self-regulation.

A total of 91.7% (494/539) of the farmers consented to participate at follow-up (Figure 6). During implementation, 91.1% (164/180) of the farmers attended both days of the educational intervention, whereas 7.8% (14/180) of the farmers were not available to attend both days. Although 91.1% (163/179) of the farmers received the educational intervention and RANAS-based SMS text messages, 8.4% (15/179) of the farmers could not receive the messages as their phones were turned off during the intervention. Preliminary results show that 84.6% (456/539) of respondents were male and 31.9% (172/539) of the farmers had received training in pesticide use before the baseline survey. The average age was 41.1 (SD 12.0) years, and 46.8% (252/539) of the farmers had attained at least secondary education. The preliminary findings are expected to be published in autumn 2024.

## Discussion

### Expected Findings

Interventions are part of the strategies for reducing pesticide exposure and the associated health consequences [17] in addition to other strategies such as using less toxic alternatives and improving the comprehensibility of hazard communication via pesticide labels [41]. This is useful especially in LMICs, where pesticide regulation enforcement is weak [42] and public extension service systems are not well supported [19]. Educational interventions aim at enlightening agricultural workers on the occupational health and environmental risks associated with pesticides and equipping them with skills with the intent to influence them to adopt safe pesticide-handling practices [15,41].

This study has several strengths, but there are also some limitations and avenues for future research. The UNACOH NGO developed their educational training curriculum over the years and is widely sensitized to the Ugandan public. This study is the first to objectively evaluate the effectiveness of these efforts through a cluster-randomized trial, assessing their ability to improve farmers’ adherence to safety precautions and the ultimate reduction in the undesirable health effects of pesticides. The holistic perspective evaluating effects on knowledge, attitudes and other psychosocial determinants, behavior, exposure, and health can be considered a strength. The results can provide evidence-based recommendations to heighten the possibilities of scalability of the UNACOH training.

Moreover, we will assess the effectiveness of an additional, more intensive approach: RANAS-based SMS text messages that target 1 behavior (PPE use) specifically. This will, on the one hand, inform about the effectiveness of such reminders and allow for first recommendations on whether targeting psychosocial determinants of behavior change can increase PPE use more than targeting farmers’ education. In addition, this study will be the first to examine the psychosocial mechanisms of interventions for safe pesticide handling. Analyzing psychosocial determinants that lead to behavior change as part of an intervention can provide essential insights into how an intervention works and can be improved. Overall, the findings of this study will support us in identifying the most efficient intervention approach.

As a further strength, the training intervention curriculum used was developed by a local NGO based on context-specific experience gained from working with local pesticide stakeholders in the country. In addition, the RANAS-based SMS text messaging intervention was developed building on previous local use experience by the same NGO, further refined by occupational health, pesticide safety, and behavior change experts and practitioners. Therefore, the intervention package used is culturally adapted to promote responsible pesticide handling among smallholder farmers in Uganda. These materials can now be used and adapted for further evidence-based educational and behavior change campaigns in similar settings (ie, other LMICs).

It is important to note that PPE use is the least effective strategy for reducing exposure, as illustrated by the hierarchy of controls, with elimination being the most effective [43]. However, PPE use remains one important focus of trainings. Given that, for the correct PPE to be used on a regular basis when applying pesticides, farmers must use the right PPE every time, this study provides valuable insights on the amount and type of training required.

### Limitations

As a limitation in sampling, it is noted that this trial only included smallholder farmers who were directly responsible for pesticide application. Significant exposure to pesticides is also expected from re-entry activities (ie, hoeing, weeding, planting, and harvesting in fields that have been treated with pesticides) and washing contaminated clothing and equipment [44]. This work is predominantly undertaken by women and girls, who face exacerbated risks because of low education and access to training on safe pesticide handling or other resources [45]. Future intervention studies urgently need to target safe pesticide practices such as PPE use during re-entry work or when handling contaminated items and explicitly target gender-equal access to training.

Our study design does not allow us to estimate whether the potentially increased effects in the SMS text messaging intervention are due to being reminded via SMS text message to handle pesticides safely and having a longer intervention than the education-only arm or to the promotion of behavioral determinants. We may partly target this limitation by investigating whether a greater number of psychosocial determinants targeted by the RANAS approach achieve intervention effects, but ideally, future intervention studies should compare educational SMS text messages with RANAS-based SMS text messages.

Finally, all our measures were self-reported, which can lead to bias regarding subjectivity and socially desirable answers. While assessments of knowledge, attitude, and other psychosocial determinants (ie, RANAS factors) are typically self-reported, there are potential ways for future studies to more objectively assess behavior and pesticide exposure. For example, they may assess (correct) PPE use through field observations or demonstrations by farmers and pesticide exposure through biomarkers in hair or urine samples. However, collecting and analyzing such data is often costly.

### Conclusions

This research will contribute to a better understanding of the effectiveness and mechanisms of educational and theory-based behavior change SMS text messaging interventions for reducing pesticide exposure among smallholder farmers. The insights from this study could be used to strategically improve and implement context-specific interventions to reduce pesticide-related risks to human and environmental health in different LMICs.

## Appendix

Multimedia Appendix 1 Baseline data collection in Uganda and intervention description, African Pesticide Intervention Project.

Multimedia Appendix 2 Educational intervention curriculum for farmers covering 5 broad topics on safe pesticide handling.

Multimedia Appendix 3 Psychosocial determinants and behavior change techniques based on Risks, Attitudes, Norms, Abilities, and Self-regulation targeted in text messages sent to farmers.

Multimedia Appendix 4 Risks, Attitudes, Norms, Abilities, and Self-regulation behavior change techniques and expected psychosocial determinants derived from the educational curriculum on safe pesticide handling.

Multimedia Appendix 5 Questionnaire.

Multimedia Appendix 6 Knowledge, attitude and practice questions along the four modules of the education curriculum on safe pesticide handling.

## Abbreviations

DFA: District Farmers’ Association
EIS: exposure intensity score
IPM: integrated pest management
KAP: knowledge, attitude, and practice
LMIC: low- and middle-income country
NGO: nongovernmental organization
ODK: Open Data Kit
PHE: Pesticide Use Health and Environment
PPE: personal protective equipment
RANAS: Risks, Attitudes, Norms, Abilities, and Self-regulation
UNACOH: Uganda National Association of Community and Occupational Health

## Data Availability

The data sets generated during and analyzed during this study are available from the corresponding author upon reasonable request.
