# Supplementary material for: Evaluating and Enhancing an Educational Intervention to Reduce Smallholder Farmers’ Exposure to Pesticides in Uganda Through a Digital, Systematic Approach to Behavior Change: Protocol for a Cluster-Randomized Controlled Trial
Source: JMIR Res Protoc. 2024 May 8;13:e55238. doi: 10.2196/55238 (PMC11112482; doi:10.2196/55238)
Supplement: Multimedia Appendix 2 [file resprot_v13i1e55238_app2.docx]

Educational intervention curriculum for farmers covering five broad topics on safe pesticide handling

| Topic (Take Home Messages) | Delivery method(s) |
| --- | --- |
| 1. Introduction to pesticides   - What are pesticides - How are pesticides are made - How are pesticides named (brand name vs. active ingredient) - How are they grouped - How pesticides work/act when applied - How does the manufacturer communicate with the user (pesticide label interpretation) - How do pests develop resistance to pesticides | - Power point presentation - Group exercices on pesticide label interpretation - Watching short videos on pesticide modes of action, resistance development |
| 2. Pesticides and human health   - Why are pesticides toxic to humans (formulation, mode of action) - How can one identify/differentiate acute toxicity levels of different pesticides (WHO colour codes on product label) - How do humans get exposed to pesticides; occupational, accidental, background (practices and routes of entry) - How to different pesticides affect human health (acute and chronic effects) - What should one do in case of direct exposure (first aid) - Who is vulnerable to exposure and effects of pesticides - How can we minimise exposure (safety precautions along the handling chain) | - Power point presentation - Viewing and discussing exposure photos from the field - Group exercises on exposure and how to minimise it - Viewing short video clips on first aid, negative health outcomes - Practical demonstration with different PPEs |
| 3. Pesticides and the environment   - Modes of pesticide movement in the environment - Sensitive/high risk areas in the environment - Effect on beneficial organisms - Protecting the environment e.g. Pesticide waste management | - Video clips on environmental pollution from pesticides - Practical demonstration of triple rinsing and puncturing of empty pesticide containers |
| 4. Pesticide application   - Pesticide application equipment (types and aspects to consider before buying a knapsack sprayer) - Understanding different sprayer parts (especially choice of nozzles for different spray jobs), cleaning and maintenance of knapsack sprayer - Calibration of Lever Operated Knapsack sprayer - Mixing pesticides (Interpretation of mixing/dosage instructions on a pesticide label and right mixing procedures) - Pesticide application techniques (to achieve precision and minimise exposure) | - Practical demonstration of mixing and calibration exercises - Viewing different sprayers and parts |
| 5. Introduction to Integrated Pest Management (IPM)   - What different approaches can be combined to manage pests and diseases - How can plants resist pests or diseases and how does this resistance develop (Host Plant Resistance) - What ecological/cultural methods can farmers use in pest/disease management - What are some of the beneficial insects in our fields that help to control pests (Biological control, predators, parasitoids) - What organic pesticides/ biopesticides can farmers use and how to access them - Trapping of insect pests using traps (light, sticky and pheromone traps) | - Practical demonstration with traps for fruit flies and fall army worm - Watching video clips |
| Evaluation of the training |  |
