# Supplementary material for: Evaluating and Enhancing an Educational Intervention to Reduce Smallholder Farmers’ Exposure to Pesticides in Uganda Through a Digital, Systematic Approach to Behavior Change: Protocol for a Cluster-Randomized Controlled Trial
Source: JMIR Res Protoc. 2024 May 8;13:e55238. doi: 10.2196/55238 (PMC11112482; doi:10.2196/55238)
Supplement: Multimedia Appendix 3 [file resprot_v13i1e55238_app3.doc]

Psychosocial determinants and behavior change techniques based on Risks, attitudes, norms, abilities and self-regulation (RANAS) targeted in text messages sent to farmers

| RANAS Psychosocial determinant | RANAS Behavior change techniques | Text message wording |
| --- | --- | --- |
| Cost-benefit | 1. Present facts  5. Inform about and assess costs and benefits  15. provide instruction | **SAFE FARMING**: Always protect your hands when handling pesticides. Chemical resistant gloves cost between 5-10,000/=. Protecting your hands from pesticides has never been cheaper!! **APSENT Project** |
| Others’ behavior and others‘ approval | 9. Inform about others’ behaviour  11. Inform about others’ approval | **SAFE FARMING**: Did you know that farmer [xx]^1^ from your training already, uses PPE and other farmers seem to be fascinated by his forward-looking attitude! Buy and use the right PPE when handling pesticides as well! **APSENT Project** |
| Attitude towards pesticide toxicity (KAP) | 1. Present facts | **SAFE FARMING**: Don’t be misguided by others. All pesticides, including herbicides can poison you. Always protect yourself. **APSENT Project** |
| Feeling (proud, not weak), *also: others’ behavior & approval* | 8. Describe feelings about performing and about consequences of the behavior  9. Inform about others’ behaviour  11. Inform about others’ approval | **SAFE FARMING**: From Atutur Subcounty, Mr. [xx]^1^ is proud of being a responsible man by protecting his health and the family’s future because he uses PPEs when handling pesticides. **APSENT Project** |
| Knowledge on best weather, Attitude (KAP) | 14. Prompt identification as role model  15. Provide instruction | **SAFE FARMING**: When you wear PPEs to apply pesticides in cool weather, you don’t feel discomfort and your neighbor may want to copy you. **APSENT Project** |
| How-to-do knowledge | 15. Provide instruction | **SAFE FARMING**: Chemical resistant gloves as well as other PPE items (glasses, overall, gumboots) that protect your body from toxic pesticides can be bought from KUDFA (phone: 0773528289) and SEDFA (0702394450) at a subsidized price for as low as Ugx 60,000/=. **APSENT Project** |
| Action plan to buy PPE (Instruction to share) | 26. Prompt specific planning | **SAFE FARMING:** Make a plan to buy all the PPE before spraying starts: Where and when do you want to cover all your body parts? Please note this plan on a paper. We will call you to hear about your plan! **APSENT PROJECT** |
| Action plan to buy PPE (Example) | 26. Prompt specific planning | **SAFE FARMING**: An example plan to buy PPE to cover all body parts: **What?**: Gloves and googles that are missing to have a complete set. **Where?** KUDFA/SEDFA **When/ How to go?** On Saturday by motorbike **APSENT Project** |
| Action plan (Example)  Others behavior about PPE ownership | 26. Prompt specific planning  9. Inform about others’ behaviour | **SAFE FARMING**: Mr. [xx]^1^ from [xx]^1^ Subcounty plans to go with his neighboring farmer on his motorbike to KUDFA/SEDFA next Saturday to buy gloves, goggles and gumboots. Think of your plan to get PPE as well! **APSENT Project** |
| Phone call 1: Action plan & Monitoring PPE purchase | 26. Prompt specific planning | The contact person will listen to/ discuss a detailed **action plan** : Where when, how to buy at least one PPE item (especially gloves)?  **Monitoring:** What have you bought so far? |
| Others behavior about PPE ownership | 9. Inform about others’ behaviour | **SAFE FARMING**: Farmer [xx]^2^ and other farmers in your area can protect his whole body during pesticide application as they bought all the relevant PPE items. Go get PPE like them! **APSENT Project** |
| Response Efficacy | 5. Inform about and assess costs and benefits | **SAFE FARMING**: As a responsible farmer, always wearing your PPE greatly reduces the chances of you getting directly contaminated with pesticides. **APSENT Project** |
| Phone call 2: Monitoring: PPE purchase, *also: action control* | 27. Prompt (self)-monitoring of behavior | Script for phone call:  What have you bought so far?  Which PPE do you use? How often? |
| Response Efficacy | 5. Inform about and assess costs and benefits | **SAFE FARMING**: Wearing the correct goggles/glasses protects your eyes from chemical contamination, thus saving you from short- and long-term vision problems. **APSENT Project** |
| Others behavior (PPE Use) | 9. Inform about others’ behaviour | **SAFE FARMING:** Majority of trained farmers in Kumi and Sembabule have become pesticide safety change agents, leading by example in using PPEs. Now is your time to join them. Take action!! **APSENT Project** |
| Response Efficacy | 5. Inform about and assess costs and benefits | **SAFE FARMING**: Wearing the correct chemical mask/respirator, especially when mixing, protects you from inhaling the chemical into your lungs thus allowing you to remain a healthy and active farmer. **APSENT Project** |
| Others behavior (PPE Use) | 9. Inform about others’ behaviour | **SAFE FARMING**: A great part of the trained farmers in Kumi and Sembabule are correctly using, cleaning and maintaining their PPEs. **APSENT Project** |
| Phone call 3: Monitoring PPE Use | 27. Prompt (self)-monitoring of behavior | Script: Which PPE do you use? How often? |
| Barrier planning | 30. Prompt coping with barriers | **SAFE FARMING**: It’s not always easy to wear PPE to cover all body parts all the time. Think of your challenges and try to develop possible solutions to ensure using of PPE every time you handle pesticides. **APSENT Project** |
| Others behavior (PPE Use) | 9. Inform about others’ behaviour | **SAFE FARMING**: Most of the trained farmers in Kumi and Sembabule have been able to start using PPEs. Have you started? **APSENT Project** |
| Barrier planning | 30. Prompt coping with barriers | **SAFE FARMING**: Some irresponsible farmers sometimes spray pesticides without PPE because they sweat and become uncomfortable. How would you advise them to overcome this challenge? **APSENT Project** |
| Others behavior (PPE Use) | 9. Inform about others’ behaviour | **SAFE FARMING**: Compared to last season, even more farmers in Kumi and Sembabule have acquired and are using PPEs this season. **APSENT Project** |
| Barrier planning | 27. Prompt (self)-monitoring of behavior  30. Prompt coping with barriers  32. Prompt to resist social pressure | **SAFE FARMING**: Are you still using PPEs? How would you respond or react to people who laugh at you for using PPEs? **APSENT Project** |

^1^Real name from peer farmer from the educational training was entered

^2^Information based on monitoring findings from phone calls was entered
