# Supplementary material for: Evaluating and Enhancing an Educational Intervention to Reduce Smallholder Farmers’ Exposure to Pesticides in Uganda Through a Digital, Systematic Approach to Behavior Change: Protocol for a Cluster-Randomized Controlled Trial
Source: JMIR Res Protoc. 2024 May 8;13:e55238. doi: 10.2196/55238 (PMC11112482; doi:10.2196/55238)
Supplement: Multimedia Appendix 4 [file resprot_v13i1e55238_app4.doc]

Risks, attitudes, norms, abilities and self-regulation (RANAS) behavior change techniques and expected psychosocial determinants derived from the education curriculum on safe pesticide handling

*Note:* This is a retrospective description and mapping of psychosocial determinants based on the educational curriculum. The curriculum was not developed based on the Ranas approach.

| **Training module** | **Activity** | **Source** | **RANAS Behavior change technique** | **Expected RANAS factor** |
| --- | --- | --- | --- | --- |
| **Module 1: Introduction to synthetic pesticides** | Farmers write up experiences with pesticide use, afterwards examples of write up are read out to other farmers. Experiences refer to:  -Different areas/use of pesticides in their homes  -Name of pesticide on the label they he/she has been given  -5 different messages from the label he/she has been given | PPT_Modul1_1, p. 5-19 | 3. Inform about and assess personal risk  27. Prompt (self)-monitoring of behavior  7. Prompt to talk to others  9. Inform about others’ behaviour | Vulnerability  Beliefs about costs and benefits  Others’ behavior |
|  | Expert presents: Pictures of pesticide products and their ingredients, groups of pesticides according to targeted pests, toxicity, mode of actions (the chemicals through which they work), pesticide life cycle and legal regulations for pesticides  Video is presented how pesticides work (on insects and fungal diseases) | PPT_Modul1_1, 20-56  PPT_Modul1_1, 53 | 1. Present facts | Health knowledge |
|  | Expert explains different pesticide product label/pictograms that indicate its toxicity and recommendations for use (e.g. only use with face mask, do not bring in contact with animals) | PPT_Modul1_1, p.27, 48 | 15. Provide instruction | How-to-do Knowledge |
|  | Expert explains what pesticide resistance means, how it develops and how to manage it (e.g. monitor pests, time applications correctly, mix and apply carefully (dose, coverage), alternate different insecticide classes)  Video is presented how pesticide resistance develops (in pests) | PPT_Modul1_2 | 1. Present facts  15. Provide instruction | Health knowledge  How-to-do  Knowledge |
| **Module 2: Pesticides and human safety** | Expert informs farmers about different toxicity groups and poison in pesticides | PPT_Modul2, p.2-8 | 1. Present facts | Health Knowledge |
|  | Expert informs farmers that exposure to pesticides can lead to death  Expert informs about forms of direct pesticide exposure (e.g. application, preparation, post-application) | PPT_Modul2, p.9 PPT_Modul2, p.11 | 2. Present scenarios:  4. Arouse fear | Health Knowledge  Severity (Vulnerability) |
|  | Group work: Farmers identify unsafe pesticide handling practices in picture (different situation of direct exposure such as person transporting bottles with pesticides in a basket with food) and suggest alternative, safer practices | PPT_Modul2, p.12-18 | 2. Present Scenario  Provide instruction | Health Knowledge  How-to-do  knowledge |
|  | Expert presents the contamination of food, water and air as an indirect exposure to pesticides (chemicals) and how these were detected in farmers bodies, e.g. in their urine and hair assessments. | PPT_Modul2, p.19-41 | 1. Present facts | Health knowledge |
|  | Expert presents short- and long-term health effects of pesticides | PPT_Modul2, p.42-56 | 1. Present facts | Health knowledge |
|  | Expert presents scenarios of indirect exposure (e.g. mixing, loading sprayers) and draws attention to that these are also undertaken by other family members, particularly children.  Experts outlines vulnerability of children related to pesticide exposure | PPT_Modul2, p.57-67 | 1. Present facts  2. Present scenarios  3. Inform about and assess personal risk | Health knowledge  Vulnerability |
|  | Group discussion: How can they (the farmers) be protected from exposure? | PPT_Modul2, p.68 | 7. Prompt to talk to others  15. Provide instruction | Beliefs about costs and benefits  How-to-knowledge |
| **Module 3: Pesticides and environmental safety** | Expert presents fate of pesticides in the environment- degradation (microbial, chemical, photo) and transfer (drift, leaching, adsorption, absorption, runoff).Expert guides brainstorming session on aspects that need special attention/protection from pesticides (bees, fish, beneficial organisms, wildlife, water)  Group discussion: How can they (the farmers) protect the environment from pesticide contamination? | PPT_Modul3, p. 2-6; 11-44  PPT_Modul3, p. 7-9  PPT_Modul3, p. 45 | 1. Present facts  2. Present scenarios  7. Prompt to talk to others | Health knowledge  Beliefs about costs and benefits |
| **Module 4:** **Pesticide Application: Equipment & Techniques** | Farmers share their examples and experiences on common pesticide formulations, commonly applied pesticides, commonly used application equipment and cleaning of application equipment | PPT_Modul4, p. 2-5, p. 32 | 15. Provide instruction  7. Prompt to talk to others  9. Inform about others’ behaviour | How-to-do-knowledge  Others’ behavior |
|  | Expert shows photos of and presents different pesticide application equipment and their advantages (e.g. easy splash-free water filling) and disadvantages (e.g. less durable) | PPT_Modul4, p.7-31 | 5. Inform about and assess costs and benefits | Beliefs about costs and benefits |
|  | Expert gives instructions on the calibration of pesticide application equipment and the calculation of pesticide doses | PPT_Modul4, p.33 -40, p.46-47 | 15. Provide instruction | How-to-do-knowledge |
|  | Expert presents fictive scenario of a farmer and instructs the farmers to calculate pesticide dose | PPT_Modul4, p.42 | 18. Prompt guided practice | Confidence in performance |
|  | The farmers practice the calibration and pesticide calculation (practical exercise outside) | PPT_Modul4, p. 45 | 18. Prompt guided practice  19. Prompt behavioral practice | Confidence in performance |
| **Module 5: Introduction to Integrated Pest Management (IPM)** | Farmers form groups and write down their known pest control strategies. The farmers select a leader to present the group strategies to the rest of the farmers. | PPT_Modul5, p. 73-82 | 7. Prompt to talk to others  9. Inform about others’ behaviour  14. Prompt identification as role model  35. Prompt goal setting | Others’ behavior  Personal importance  Commitment |
|  | An expert (Government extension agent) expounds on the importance and the various control strategies that can be used in order to reduce pesticide reliance. |  | 1. Present facts  15. Provide instruction  11. Inform about others’ approval / disapproval | Health knowledge How-to-do-knowledge  Others’ approval |
