# Supplementary material for: Evaluating and Enhancing an Educational Intervention to Reduce Smallholder Farmers’ Exposure to Pesticides in Uganda Through a Digital, Systematic Approach to Behavior Change: Protocol for a Cluster-Randomized Controlled Trial
Source: JMIR Res Protoc. 2024 May 8;13:e55238. doi: 10.2196/55238 (PMC11112482; doi:10.2196/55238)
Supplement: Multimedia Appendix 5 [file resprot_v13i1e55238_app5.pdf]

Swiss TPH
Swiss Tropical and Public Health Institute

Vital Strategies

Bloomberg Philanthropies

DATA FOR HEALTH INITIATIVE

Survey

apsent_ug_endline_v6

Available Languages	English, Luganda, Ateso, Runyakitara.

[start] [start]

[end] [end]

[today] [today]

[deviceid] [deviceid]

[time_start] [time]
Please take the time

[ ] [note]
#Beginning

[ent] [select_one]

1) Interview conducted by

[1]	Peter Ssekkadde
[2]	Joseph Walusimbi
[3]	Acom Gorretti
[4]	Vencia Naggayi
[5]	Bernard Twinamatsiko
[6]	Charles Apunyo
[7]	Pamela Musimenta
[8]	Anthony Emaru
[9]	Gerald Ilukor
[10]	Ruth Ikwangat
[11]	Ronnet Ayebare
[12]	Nicholas Oluka
[13]	Kassim Tegawa
[14]	Aggrey Atuhaire
[15]	Samuel Fuhrimann
[16]	Curdin Brugger
[17]	Vica Tomberge
[96]	Other

• Relevant when:
1) Interview conducted by was answered with Other.

[ent_sp] [text]
Specify other

[language] [select_one]
1.1) What language was the interview conducted in?

[1]	English
[2]	Luganda
[3]	Runyakitara
[4]	Ateso

[district_1] [select_one]
2) District

[1]	Sembabule
[2]	Kumi

• Relevant when:
2) District was answered with Sembabule.

[subcounty_s_1] [select_one]
3) Subcounty

[7]	Lugusuulu
[8]	Lwebitakuli
[9]	Lwemiyaga
[10]	Mateete
[11]	Mijwala
[12]	Ntuusi

• Relevant when:
2) District was answered with Kumi.

[subcounty_k_1] [select_one]
3) Subcounty

[1]	Atutur
[2]	Kanyum
[3]	Kumi
[4]	Mukongoro
[5]	Nyero
[6]	Ongino

• Relevant when:
3) Subcounty was answered with Atutur.

[id_k1] [integer]
4) ID Number of the farmer

• Relevant when:
3) Subcounty was answered with Kanyum.

[id_k2] [integer]
4) ID Number of the farmer

• Relevant when:
3) Subcounty was answered with Kumi.

[id_k3] [integer]
4) ID Number of the farmer

• Relevant when:
3) Subcounty was answered with Mukongoro.

[id_k4] [integer]
4) ID Number of the farmer

• Relevant when:
3) Subcounty was answered with Nyero.

[id_k5] [integer]
4) ID Number of the farmer

• Relevant when:
3) Subcounty was answered with Ongino.

[id_k6] [integer]
4) ID Number of the farmer

• Relevant when:
3) Subcounty was answered with Lugusuulu.

[id_s7] [integer]
4) ID Number of the farmer

• Relevant when:
3) Subcounty was answered with Lwebitakuli.

[id_s8] [integer]
4) ID Number of the farmer

• Relevant when:
3) Subcounty was answered with Lwemiyaga.

[id_s9] [integer]
4) ID Number of the farmer

• Relevant when:
3) Subcounty was answered with Mateete.

[id_s10] [integer]
4) ID Number of the farmer

• Relevant when:
3) Subcounty was answered with Mijwala.

[id_s11] [integer]
4) ID Number of the farmer

• Relevant when:
3) Subcounty was answered with Ntuusi.

[id_s12] [integer]
4) ID Number of the farmer

[consent] [select_one]
5) Does the farmer agree to take part in the survey?

[1]	yes
[0]	no

• Relevant when:
5) Does the farmer agree to take part in the survey? was answered with no.

[consent_no] [text]
5.1) What is the reason the farmer does not take part in the survey?

• Relevant when:
5) Does the farmer agree to take part in the survey? was answered with no.

[ ] [note]
The interview with this farmer is done. Please still save the questionnaire and upload the data to the server even though the interview was not completed.

• Relevant when:
5) Does the farmer agree to take part in the survey? was answered with yes.

[sex] [select_one]
6) Gender

[1]	male
[2]	female

• Relevant when:
5) Does the farmer agree to take part in the survey? was answered with yes.

[intervention] [select_one]
7) To which intervention group did the participant belong?

[1]	Control (no intervention)
[2]	2-day training
[3]	2-day training + SMS

• Relevant when:
5) Does the farmer agree to take part in the survey? was answered with yes.

[ ] [note]
Make sure that you started the voice recording

• Relevant when:
5) Does the farmer agree to take part in the survey? was answered with yes.

[ ] [note]
# Questionnaire

• Relevant when:
5) Does the farmer agree to take part in the survey? was answered with yes.

[time_b] [time]
Please take the time

• Relevant when:
5) Does the farmer agree to take part in the survey? was answered with yes.

[ ] [note]
Lets continue now with a structured questionnaire where we would like to learn more about your personal and farming characteristics. We then continue with specific questions around your knowledge, attitudes and practices of pesticide use and finaly we will ask you some questions about your health. Some of these questsions are personal and you may just let us know if you do not want to respond.

• Relevant when:
5) Does the farmer agree to take part in the survey? was answered with yes.

[ ] [note]
#B. Lets start with your personal characteristics

• Relevant when:
5) Does the farmer agree to take part in the survey? was answered with yes.

B. Respondents' personal characteristics

[age] [integer]
10) How old are you?

[smo_ac] [select_one]
11) Do you currently smoke tobacco?

[0]	No
[1]	Yes
[98]	Don't know

[mar_ac] [select_one]
12) Do you currently smoke marijuana?

[0]	No
[1]	Yes
[98]	Don't know

[alc_b] [select_one]

13) Do you currently drink alcoholic beverages or liquor?

[0]	No
[1]	Yes
[98]	Don't know

[drug1] [select_one]

14) Do you consume any other recreational drugs?

[0]	No
[1]	Yes
[98]	Don't know

[occ_main] [select_one]

15) What is your main occupation?

[1]	Farmer
[2]	Nurse / pharmacist / doctor / veterinary doctor
[3]	Extension worker
[4]	Agro-input dealer
[5]	Fisher
[6]	Mechanic / Builder / Lumber Jack
[7]	Police Officer
[8]	Business owner
[9]	Teacher
[96]	Other

- Relevant when:

15) What is your main occupation? was answered with Other.

[occ_main_sp] [text]

15.1) Specify main job

[hou_mo] [select_one]

16) What is the average monthly income for your family? Please include everyone who contributes to the income of the household: Household head, partner, children, relatives etc.

UGX / per month

[1]	less then 50,000
[2]	50,001 and 100,000
[3]	100'001 and 500,000
[4]	500,001 and 1,000,000
[5]	1,000,001 and 1,500,000
[6]	1,500,001 and 2,000,000
[7]	2,000,001 and 2,500,000
[8]	2,500,001 and 3,000,000
[9]	3,000,001 and 3,500,000
[10]	3,500,001 and 4,000,000
[11]	4,000,001 and 4,500,000
[12]	4,500,001 and 5,000,000
[97]	don't know
[98]	not willing to respond

[hou_agr] [integer]

16.1) Of the average monthly income, what proportion is from the family farming enterprises?

write % 0 and 100

[inc_pe] [integer]

16.2) How many people, including you, live off this income?

This includes not only the people that live in your house, but also other people receiving parts of this money.

[hou_mo_coivid] [select_one]

16.3) To what extend was the income affected by COVID in the past year?

[1]	Covid not affected the monthly income
[2]	Increased slightly (up to 25% more the usuall)
[3]	Increased considerably (more then 25% more the usuall)
[4]	Decreased slightly (up to 25% more the usuall)
[5]	Decreased considerably (more then 25% more the usuall)

• Relevant when:
5) Does the farmer agree to take part in the survey? was answered with yes.

[ ] [note]
Thanks for answering to these questions, now we would like to learn more about your farming characteristics

[ ] [note]
#C. Respondents' farming characteristics
We are now going to talk about some characteristics of the house you live in and the farm you work in.

• Relevant when:
5) Does the farmer agree to take part in the survey? was answered with yes.

[time_c] [time]
Please take the time

• Relevant when:
5) Does the farmer agree to take part in the survey? was answered with yes.

C. Respondents' farming characteristics

[farm_ob] [select_one]

20) What is your main farming objective?

[1]	subsistence
[2]	commercial

[wofo] [decimal]

21) Including you, how many people work on these farms in total?

If there are people working under the age of 18 they count as 0.5 98 = don't know

[crop1] [select_one]

22) What is the first major crop you are _currently_ growing?

[38]	Apple
[36]	Avocado
[19]	Bananas
[4]	Beans
[6]	Cabbage
[14]	Carrot
[22]	Cassava
[8]	Coffee
[28]	Cotton
[16]	Cow peas
[17]	Cucumber
[1]	Egg plants
[34]	Eucalyptus
[2]	Flowers
[21]	Grass
[11]	Green Gram
[5]	Ground nuts
[15]	Maize
[20]	Mangoes
[40]	Millet

[23]	Nakati
[25]	Onion
[7]	Oranges
[24]	Passion fruit
[27]	Paw Paw
[13]	Pepper
[44]	Pineapple
[43]	Potato (Irish)
[18]	Potato (Sweet)
[35]	Red pepper
[39]	Rice
[9]	Sim Sim
[41]	Sorghum
[42]	Soya bean
[29]	Spinach
[32]	Sugar cane
[30]	Sukuma
[45]	Sunflower
[46]	Tobacco
[10]	Tomato
[37]	Vanilla
[31]	Water melon

[70]	Crop not specified
[96]	Other (specify)

• Relevant when:
22) What is the first major crop you are _currently_ growing? was answered with Other (specify).

[crop1_sp] [text]
22.1) Specify other

[crop2] [select_one]

23) What is the second major crop you are _currently_ growing?

[38]	Apple
[36]	Avocado
[19]	Bananas
[4]	Beans
[6]	Cabbage
[14]	Carrot
[22]	Cassava
[8]	Coffee
[28]	Cotton
[16]	Cow peas
[17]	Cucumber
[1]	Egg plants
[34]	Eucalyptus
[2]	Flowers
[21]	Grass
[11]	Green Gram
[5]	Ground nuts
[15]	Maize
[20]	Mangoes
[40]	Millet

[23]	Nakati
[25]	Onion
[7]	Oranges
[24]	Passion fruit
[27]	Paw Paw
[13]	Pepper
[44]	Pineapple
[43]	Potato (Irish)
[18]	Potato (Sweet)
[35]	Red pepper
[39]	Rice
[9]	Sim Sim
[41]	Sorghum
[42]	Soya bean
[29]	Spinach
[32]	Sugar cane
[30]	Sukuma
[45]	Sunflower
[46]	Tobacco
[10]	Tomato
[37]	Vanilla
[31]	Water melon

[70]	Crop not specified
[96]	Other (specify)

• Relevant when:
23) What is the second major crop you are _currently_ growing? was answered with Other (specify).

[crop2_sp] [text]
23.1) Specify other

[crop3] [select_one]

24) What is the third major crop you are _currently_ growing?

[38]	Apple
[36]	Avocado
[19]	Bananas
[4]	Beans
[6]	Cabbage
[14]	Carrot
[22]	Cassava
[8]	Coffee
[28]	Cotton
[16]	Cow peas
[17]	Cucumber
[1]	Egg plants
[34]	Eucalyptus
[2]	Flowers
[21]	Grass
[11]	Green Gram
[5]	Ground nuts
[15]	Maize
[20]	Mangoes
[40]	Millet

[23]	Nakati
[25]	Onion
[7]	Oranges
[24]	Passion fruit
[27]	Paw Paw
[13]	Pepper
[44]	Pineapple
[43]	Potato (Irish)
[18]	Potato (Sweet)
[35]	Red pepper
[39]	Rice
[9]	Sim Sim
[41]	Sorghum
[42]	Soya bean
[29]	Spinach
[32]	Sugar cane
[30]	Sukuma
[45]	Sunflower
[46]	Tobacco
[10]	Tomato
[37]	Vanilla
[31]	Water melon

[70]	Crop not specified
[96]	Other (specify)

• Relevant when:
24) What is the third major crop you are _currently_ growing? was answered with Other (specify).

[crop3_sp] [text]
24.1) Specify other

[pest_control] [select_multiple]

25) What are the _three_ main pest management approaches that you use

read out loud, multiple answers possible, select only three options

[1]	Manual weeding
[2]	Pesticide use
[3]	Using resistant/tolerant varieties
[4]	Intercropping
[5]	Early planting
[6]	Crop rotation
[7]	Push-pull technology
[8]	Mulching
[9]	Field sanitation
[10]	Bio pesticide
[11]	Hand picking
[12]	Rouging
[13]	Manure use
[14]	Fallowing
[15]	Organic
[16]	Physical/manual/mechanical killing
[17]	Prunning
[18]	Scouting
[19]	Spacing

[20]	Synthetic fertiliser use
[21]	Thinning
[22]	Burning
[23]	Trapping
[96]	Other
[99]	Nothing

• Relevant when:
25) What are the _three_ main pest management approaches that you use was answered with Other.

[pest_control_sp] [text]
25.1) Specify other

[challange_coivd_neg] [select_multiple]

26) How did the COVID-19 situation negatively impact your farming in the past months?

read out loud, multiple answers possible

[1]	Decrease in income
[2]	Lack of market for farm products
[3]	Reduced labour
[4]	Limited access to transport
[5]	Limited access to agro inputs
[6]	Limited access to agricultural extension services
[7]	Fall in prices of farm produce
[8]	More expensive labour
[9]	More expensive agro inputs
[10]	Higher cost
[11]	More fear / stress
[12]	Less contact with community / farmers
[13]	More crime
[96]	Other
[99]	None

• Relevant when:
26) How did the COVID-19 situation negatively impact your farming in the past months? was answered with Other.

[challange_coivd_neg_sp] [text]
26.1) Other negatively impact faced due to COVID-19?

[challange_coivd] [select_multiple]

27) How did the COVID-19 situation positively impact your farming in the past months?

read out loud, multiple answers possible

[1]	Increase in income
[2]	Increase of market for farm products
[3]	increased labour
[4]	increased access to transport
[5]	Increased access to agro inputs
[6]	Increased access to agricultural extension services
[7]	Increase in prices of farm produce
[8]	Financial support from government and NGOs
[9]	More time to do working on the field
[10]	Cheap labour
[11]	Improvement in farming skills

[12]	Increased acreage
[13]	Increased food
[14]	Lower cost / saved money
[15]	More family time
[16]	Appreciation of occupation
[96]	Other
[99]	None

• Relevant when:
27) How did the COVID-19 situation positively impact your farming in the past months? was answered with Other.

[challange_coivd_sp] [text]
27.1) Other positive impact faced due to COVID-19

[tra_ye] [integer]
28) How old were you when you started working on agricultural farms?
__ *age*

[pe_sy_ag] [integer]

29) At what age did you start mixing or applying synthetic pesticides?

__ *age*

[who1] [select_multiple]

30) Who mixes the pesticides you are using?

Read out loud, select multiple

[1]	Me
[2]	Maintanance aid or washerwoman of the farm
[3]	Someone I hire occasionally
[4]	Someone I employ regularly
[5]	Partner
[6]	Father
[7]	Brother
[8]	Son
[9]	Mother
[10]	Sister
[11]	Daughter
[12]	Other relatives
[13]	Whoever mixes / uses pesticide
[77]	Nobody (They aren't washed / disposed)

[96]	Other
[98]	No response given

• Relevant when:
30) Who mixes the pesticides you are using? was answered with Other.

[who1_sp] [text]
30.1) Specify other

• Relevant when:
6) Gender was answered with female.

[ppe_preg1] [select_one]
31) Do you apply or mix pesticides when you are being pregnant?

[1]	Yes
[0]	No
[9]	I have never been pregnant
[98]	No response given

[who2] [select_multiple]

32) Who disposes your pesticide containers?

Read out loud, select multiple

[1]	Me
[2]	Maintanance aid or washerwoman of the farm
[3]	Someone I hire occasionally
[4]	Someone I employ regularly
[5]	Partner
[6]	Father
[7]	Brother
[8]	Son
[9]	Mother
[10]	Sister
[11]	Daughter
[12]	Other relatives
[13]	Whoever mixes / uses pesticide
[77]	Nobody (They aren't washed / disposed)
[96]	Other
[98]	No response given

• Relevant when:
32) Who disposes your pesticide containers? was answered with Other.

[who2_sp] [text]
32.1) Specify other

[who3] [select_multiple]

33) Who washes your pesticide application equipment?

Read out loud, select multiple

[1]	Me
[2]	Maintanance aid or washerwoman of the farm
[3]	Someone I hire occasionally
[4]	Someone I employ regularly
[5]	Partner
[6]	Father
[7]	Brother
[8]	Son
[9]	Mother
[10]	Sister
[11]	Daughter
[12]	Other relatives
[13]	Whoever mixes / uses pesticide
[77]	Nobody (They aren't washed / disposed)
[96]	Other
[98]	No response given

• Relevant when:
33) Who washes your pesticide application equipment? was answered with Other.

[who3_sp] [text]
33.1) Specify other

[who4] [select_multiple]

34) Who works in the field within 24 hours after pesticide application?

Read out loud, select multiple means the same day or the day after the pesticide was sprayed

[1]	Me
[2]	Maintanance aid or washerwoman of the farm
[3]	Someone I hire occasionally
[4]	Someone I employ regularly
[5]	Partner
[6]	Father
[7]	Brother
[8]	Son
[9]	Mother
[10]	Sister
[11]	Daughter
[12]	Other relatives
[13]	Whoever mixes / uses pesticide
[77]	Nobody (They aren't washed / disposed)
[96]	Other
[98]	No response given

• Relevant when:
34) Who works in the field within 24 hours after pesticide application? was answered with Other.

[who4_sp] [text]
34.1) Specify other

[cap_source] [select_multiple]

35) What are your three main sources of _farming_ information?

select only three

[1]	Farmer groups / associations
[2]	Neighbours / Fellow farmers / Friends
[3]	Family / Relatives
[4]	Community leaders
[5]	Extension worker
[6]	Agro-Input Dealer
[7]	NGOs
[8]	Pesticide and seed companies
[9]	Radio
[10]	Newspaper
[11]	Television
[12]	Mobile Phone / Text messages
[14]	Religious leader
[15]	Literature
[16]	Academic institution
[17]	Personal experience
[18]	Health facility
[19]	Internet
[20]	Training

[96]	Other
[99]	None

• Relevant when:
35) What are your three main sources of _farming_ information? was answered with Other.

[cap_source_sp] [text]
35.1) Specify other

[cap_source_pest] [select_multiple]

36) What are your three main sources of information on _pesticide use_?

select only three

[1]	Farmer groups / associations
[2]	Neighbours / Fellow farmers / Friends
[3]	Family / Relatives
[4]	Community leaders
[5]	Extension worker
[6]	Agro-Input Dealer
[7]	NGOs
[8]	Pesticide and seed companies
[9]	Radio
[10]	Newspaper
[11]	Television
[12]	Mobile Phone / Text messages
[14]	Religious leader
[15]	Literature
[16]	Academic institution
[17]	Personal experience
[18]	Health facility
[19]	Internet
[20]	Training

[96]	Other
[99]	None

• Relevant when:
36) What are your three main sources of information on _pesticide use_? was answered with Other.

[cap_source_pest_sp] [text]
36.1) Specify other

• Relevant when:
7) To which intervention group did the participant belong? was answered with Control (no intervention).

[cap_1] [select_one]
37) Have you been trained since the last visit on how to apply pesticides?

[0]	No
[1]	Yes
[98]	Don't know

• Relevant when:
7) To which intervention group did the participant belong? was answered with 2-day training OR 7) To which intervention group did the participant belong? was answered with 2-day training + SMS.

[cap_2] [select_one]
37) Have you been trained since the last visit on how to apply pesticides apart from the training we provided?

[0]	No
[1]	Yes
[98]	Don't know

• Relevant when:
37) Have you been trained since the last visit on how to apply pesticides? was answered with Yes OR 37) Have you been trained since the last visit on how to apply pesticides apart from the training we provided? was answered with Yes.

[cap_qu] [select_multiple]
37.1) Who trained you?

[1]	Fellow Farmer
[2]	Government
[3]	NGO
[4]	if NGO was it Uganda National Association of Community and Occupational Health (UNACOH)
[5]	CBO
[6]	Private company
[7]	Academic institution
[8]	Agriculturist / Extension worker
[9]	Family / Relative
[96]	Other
[98]	Don't know

• Relevant when:
37.1) Who trained you? was answered with Other.

[cap_qu_sp] [text]
37.2) Specify other

[train_ppe] [select_one]
38) Were you trained on using PPE?

[1]	yes
[0]	no

[covid] [select_one]
39) Have you got information/training on COVID so far?

[0]	No
[1]	Yes
[98]	Don't know

• Relevant when:
39) Have you got information/training on COVID so far? was answered with Yes.

[covid_qu] [text]
39.1) Who provided the information on COVID?

• Relevant when:
39.1) Who provided the information on COVID? was answered with 96.

[covid_qu_sp] [text]
39.2) Specify other

[covid_hyg] [select_multiple]

40) Did you change your hygienic praticides due to Covid in the past year? If yes, which ones?

Read out loud the options

[1]	No chages
[2]	Wearing face mask
[3]	Washing hands more often
[4]	Sanitizing hands with alcohol or similar liquids more often
[5]	Avoiding large crowds
[6]	Avoiding public transport
[96]	Other changes in hygenic behaviour/control measures

• Relevant when:

40) Did you change your hygienic praticides due to Covid in the past year? If yes, which ones? was answered with Other changes in hygenic behaviour/control measures.

[covid_hyg_sp] [text]

40.1) Specifiy other hygienic practises or protection you adapted?

[covid_hyg_n] [select_multiple]

41) Is there hygienic practises or protection against Covid you would like to adapt but you can not?

[1]	No chages
[2]	Wearing face mask
[3]	Washing hands more often
[4]	Sanitizing hands with alcohol or similar liquids more often
[5]	Avoiding large crowds
[6]	Avoiding public transport
[96]	Other changes in hygenic behaviour/control measures

• Relevant when:

41) Is there hygienic practises or protection against Covid you would like to adapt but you can not? was answered with Other changes in hygenic behaviour/control measures.

[covid_hyg_n_sp] [text]

41.1) Specifiy other hygienic practises or protection you would like to adapt?

• Relevant when:
5) Does the farmer agree to take part in the survey? was answered with yes.

[ ] [note]
Now let us talk a little bit about hygienic measures around pesticide use.

• Relevant when:
5) Does the farmer agree to take part in the survey? was answered with yes.

Hygiene behaviour

[wash] [select_one]
44) How long after you applied pesticides do wash your hands?
read out all the options

[1]	Immediately after (within a hour)
[2]	A few hours later (1 to 4 hours later)
[3]	Many hours later (more the 4 hours later on the same day)
[4]	The next day or later
[77]	Never
[98]	No response given

[bath] [select_one]

45) How long after you applied pesticides do you ususally take a bath or shower?

read out all the options

[1]	Immediately after (within a hour)
[2]	A few hours later (1 to 4 hours later)
[3]	Many hours later (more the 4 hours later on the same day)
[4]	The next day or later
[77]	Never
[98]	No response given

[clothes2] [select_one]

46) How long after you applied pesticides do you change your clothes?

read out all the options

[1]	Immediately after (within a hour)
[2]	A few hours later (1 to 4 hours later)
[3]	Many hours later (more the 4 hours later on the same day)
[4]	The next day or later
[77]	Never
[98]	No response given

- Relevant when:

5) Does the farmer agree to take part in the survey? was answered with yes.

Health

[enter] [select_multiple]

47) Through which body parts do you think pesticides can enter the human body?

read out loud

[77]	Pesticides can not enter the body
[1]	Nose (inhalation)
[2]	Skin (dermal)
[3]	Mouth (ingestion)
[4]	Eyes (mucous membranes)
[5]	pesticides can enter through all body parts
[6]	Eating of the crops
[7]	(Finger) nails
[96]	Other
[98]	Don't know/none mentioned

• Relevant when:
47) Through which body parts do you think pesticides can enter the human body? was answered with Other.

[enter_other] [text]
47.1) If other, please specify.

[symp] [select_multiple]

48) What are potential consequences of pesticide use (through inhalation and dermal transmission)?

[1]	Nausea
[2]	Blurred vision
[3]	Dizziness
[4]	Salivation
[5]	Skin irritation
[6]	Muscular weakness
[7]	Headache
[8]	Trembling hands
[9]	Respiratory difficulties
[10]	Extreme tiredness
[11]	Vomiting
[12]	Abdominal pain
[13]	Loss of appetite

[14]	Lack of coordination
[15]	Excessive sweating
[16]	Speech difficulty
[17]	Dry mouth / throat
[18]	Back pain
[19]	Body odour
[20]	Ear pain
[21]	Feeling hungry
[22]	Flu
[23]	Genital itching
[24]	Hardening and peeling of hands
[25]	Heart problems
[26]	Itchy / teary eyes
[27]	Runny or painful nose
[28]	Joint pain
[29]	Male infertility
[30]	Mental problems
[31]	Sneezing
[32]	Thirst
[55]	Long term effects
[66]	Death
[96]	Other

[98]	Don't know/none mentioned

• Relevant when:
48) What are potential consequences of pesticide use (through inhalation and dermal transmission)? was answered with Other.

[symp_other] [text]
48.1) If other, please specify.

• Relevant when:
5) Does the farmer agree to take part in the survey? was answered with yes.

[ ] [note]
# Please save the questionnaire

• Relevant when:
5) Does the farmer agree to take part in the survey? was answered with yes.

[ ] [note]
#D. We will continue with questions about your knowledge, attitudes and practice of towards pesticide use. Just try to answer the questions as good as you can

• Relevant when:
5) Does the farmer agree to take part in the survey? was answered with yes.

[time_d] [time]
Please take the time

• Relevant when:
5) Does the farmer agree to take part in the survey? was answered with yes.

[ ] [note]
Some of the following questions are wrong and some are right. Please answer the following questions according to the best of your KNOWLEDGE with true, false or not sure.

• Relevant when:
5) Does the farmer agree to take part in the survey? was answered with yes.

[k1] [select_one]
50) The name of the pesticide active ingredients can be found on the label of the product

[1]	True
[2]	False
[3]	Not sure

• Relevant when:
5) Does the farmer agree to take part in the survey? was answered with yes.

[k2] [select_one]
51) The pesticide label contains relevant information on how to handle and use the product

[1]	True
[2]	False
[3]	Not sure

• Relevant when:
5) Does the farmer agree to take part in the survey? was answered with yes.

[k3] [select_one]

52) The dose recommended on the label is only a suggestion and should be adapted by the farmer according to needs.

[1]	True
[2]	False
[3]	Not sure

• Relevant when:
5) Does the farmer agree to take part in the survey? was answered with yes.

[k4] [select_one]

53) When one pesticide is used frequently, pests get used to it and develop resistanc

[1]	True
[2]	False
[3]	Not sure

• Relevant when:
5) Does the farmer agree to take part in the survey? was answered with yes.

[k5] [select_one]

54) Empty pesticide containers should never be reused for packing any food stuff at home

[1]	True
[2]	False
[3]	Not sure

• Relevant when:
5) Does the farmer agree to take part in the survey? was answered with yes.

[k6] [select_one]

55) By smelling on a pesticide, one can tell how toxic/hazardous it is

[1]	True
[2]	False
[3]	Not sure

• Relevant when:
5) Does the farmer agree to take part in the survey? was answered with yes.

[k7] [select_one]

56) Pesticides have negative effects on the health of children

[1]	True
[2]	False
[3]	Not sure

• Relevant when:
5) Does the farmer agree to take part in the survey? was answered with yes.

[k8] [select_one]

57) One can only get exposed to pesticides during spraying

[1]	True
[2]	False
[3]	Not sure

• Relevant when:
5) Does the farmer agree to take part in the survey? was answered with yes.

[k9] [select_one]

58) Pesticides travel in space and pollute the air we breathe in

[1]	True
[2]	False
[3]	Not sure

• Relevant when:
5) Does the farmer agree to take part in the survey? was answered with yes.

[k10] [select_one]

59) Leaving a distance of at least 5 meters between your garden and a nearby water way is a responsible practice

[1]	True
[2]	False
[3]	Not sure

• Relevant when:
5) Does the farmer agree to take part in the survey? was answered with yes.

[k11] [select_one]
60) Pesticides which kill all the insects in the field are the most effective

[1]	True
[2]	False
[3]	Not sure

• Relevant when:
5) Does the farmer agree to take part in the survey? was answered with yes.

[k12] [select_one]
61) Pesticide containers can be discharged with the normal waste

[1]	True
[2]	False
[3]	Not sure

• Relevant when:
5) Does the farmer agree to take part in the survey? was answered with yes.

[k13] [select_one]

62) Checking your spraying equipment for any leakages before mixing is a good practice

[1]	True
[2]	False
[3]	Not sure

• Relevant when:
5) Does the farmer agree to take part in the survey? was answered with yes.

[k14] [select_one]

63) The best time to spray is during hot/sunny times of the day

[1]	True
[2]	False
[3]	Not sure

• Relevant when:
5) Does the farmer agree to take part in the survey? was answered with yes.

[k15] [select_one]

64) It is a good practice to have one spraying equipment which can do all kinds of spraying including animals and crops

[1]	True
[2]	False
[3]	Not sure

• Relevant when:
5) Does the farmer agree to take part in the survey? was answered with yes.

[k16] [select_one]

65) A good sprayer should have different types of nozzles

[1]	True
[2]	False
[3]	Not sure

• Relevant when:
5) Does the farmer agree to take part in the survey? was answered with yes.

[ ] [note]
Now we would like to know what you think the following pictograms which are on pesticide bottels mean? Please provide use your best guess?

• Relevant when:
5) Does the farmer agree to take part in the survey? was answered with yes.

label / pictograms

[pic1] [select_one]
66.1) item 1 (keep locked away and out of reach of children)
cheat sheet 4 - label

[1]	correct response
[0]	false response
[98]	Don't know

[pic2] [select_one]
66.2) item 2 (wear gloves)
cheat sheet 4 - label

[1]	correct response
[0]	false response
[98]	Don't know

[pic3] [select_one]
66.3) item 3 (wear boots)
cheat sheet 4 - label

[1]	correct response
[0]	false response
[98]	Don't know

[pic4] [select_one]
66.4) item 4 (dangerous/harmful to livestock and poultry)
cheat sheet 4 - label

[1]	correct response
[0]	false response
[98]	Don't know

[pic5] [select_one]

66.5) item 5 (wear eye protection)

cheat sheet 4 - label

[1]	correct response
[0]	false response
[98]	Don't know

[pic6] [select_one]

66.6) item 6 (wear protection over nose and mouth)

cheat sheet 4 - label

[1]	correct response
[0]	false response
[98]	Don't know

[pic7] [select_one]

66.7) item 7 (dangerous/harmful to fish and water bodies)

cheat sheet 4 - label

[1]	correct response
[0]	false response
[98]	Don't know

[pic8] [select_one]

66.8) item 8 (wash hands/body after use)

cheat sheet 4 - label

[1]	correct response
[0]	false response
[98]	Don't know

[pic9] [select_one]

66.9) item 9 (wear respirator)

cheat sheet 4 - label

[1]	correct response
[0]	false response
[98]	Don't know

[pic10] [select_one]

66.10) item 10 (harmful/irritant)

cheat sheet 4 - label

[1]	correct response
[0]	false response
[98]	Don't know

[pic11] [select_one]

66.11) item 11 (wear apron)

cheat sheet 4 - label

[1]	correct response
[0]	false response
[98]	Don't know

[pic12] [select_one]

66.12) item 12 (wear overall)

cheat sheet 4 - label

[1]	correct response
[0]	false response
[98]	Don't know

[pic13] [select_one]

66.13) item 13 ((very) toxic)

cheat sheet 4 - label

[1]	correct response
[0]	false response
[98]	Don't know

• Relevant when:
5) Does the farmer agree to take part in the survey? was answered with yes.

[ ] [note]
In the following I will aim to assess how much you agree with a feeling or statement. You can use these dots to indicate your opinion. When you agree not at all, it is the small, when you aree a little it is the second, when you somewhat agree it is the third dot, when you rather agree it is this dot and when you agree very much you choose this one, the big one.
explain 5 dote scale for agreement

• Relevant when:
5) Does the farmer agree to take part in the survey? was answered with yes.

[hungry1] [select_one]
For example, please indicate, how much are you hungry at the moment?
Cheat sheet 5 - Use 5 dots scale. Read out all options.

[1]	Not hungry at all
[2]	A little hungry
[3]	Somewhat hungry
[4]	Rather hungry
[5]	Very hungry
[98]	No response given

• Relevant when:
5) Does the farmer agree to take part in the survey? was answered with yes.

[a1] [select_one]
67) It is important for me to know the active ingredients in a given pesticide
Cheat sheet 5 - Use 5 dots scale. Read out all options.

[1]	agree not at all
[2]	agree a little
[3]	somewhat agree
[4]	rather agree
[5]	strongly agree
[98]	no response given

• Relevant when:
5) Does the farmer agree to take part in the survey? was answered with yes.

[a2] [select_one]

68) It is necessary to read instructions on pesticide label

Cheat sheet 5 - Use 5 dots scale. Read out all options.

[1]	agree not at all
[2]	agree a little
[3]	somewhat agree
[4]	rather agree
[5]	strongly agree
[98]	no response given

• Relevant when:
5) Does the farmer agree to take part in the survey? was answered with yes.

[a3] [select_one]
69) Herbicides are only killing plants and are not dangerous to humans
Cheat sheet 5 - Use 5 dots scale. Read out all options.

[1]	agree not at all
[2]	agree a little
[3]	somewhat agree
[4]	rather agree
[5]	strongly agree
[98]	no response given

• Relevant when:
5) Does the farmer agree to take part in the survey? was answered with yes.

[a4] [select_one]

70) If you follow the mixing rate on the label, the pesticide doesn't not work well

Cheat sheet 5 - Use 5 dots scale. Read out all options.

[1]	agree not at all
[2]	agree a little
[3]	somewhat agree
[4]	rather agree
[5]	strongly agree
[98]	no response given

• Relevant when:
5) Does the farmer agree to take part in the survey? was answered with yes.

[a5] [select_one]

71) Pests only develop resistance to a pesticide if it is fake/counterfeit

Cheat sheet 5 - Use 5 dots scale. Read out all options.

[1]	agree not at all
[2]	agree a little
[3]	somewhat agree
[4]	rather agree
[5]	strongly agree
[98]	no response given

• Relevant when:
5) Does the farmer agree to take part in the survey? was answered with yes.

[a6] [select_one]

72) When washed well, an empty pesticide container is safe to use at home

Cheat sheet 5 - Use 5 dots scale. Read out all options.

[1]	agree not at all
[2]	agree a little
[3]	somewhat agree
[4]	rather agree
[5]	strongly agree
[98]	no response given

• Relevant when:
5) Does the farmer agree to take part in the survey? was answered with yes.

[a7] [select_one]
73) As a farmer I am not interested in knowing the hazard level of a pesticide as long as it kills the pests on my farm
Cheat sheet 5 - Use 5 dots scale. Read out all options.

[1]	agree not at all
[2]	agree a little
[3]	somewhat agree
[4]	rather agree
[5]	strongly agree
[98]	no response given

• Relevant when:
5) Does the farmer agree to take part in the survey? was answered with yes.

[a8] [select_one]
74) Involving children in mixing and applying pesticides helps to equip them with farming skills at a young age
Cheat sheet 5 - Use 5 dots scale. Read out all options.

[1]	agree not at all
[2]	agree a little
[3]	somewhat agree
[4]	rather agree
[5]	strongly agree
[98]	no response given

• Relevant when:
5) Does the farmer agree to take part in the survey? was answered with yes.

[a9] [select_one]

75) I am comfortable working in the field on the same day that I spray it

Cheat sheet 5 - Use 5 dots scale. Read out all options.

[1]	agree not at all
[2]	agree a little
[3]	somewhat agree
[4]	rather agree
[5]	strongly agree
[98]	no response given

• Relevant when:
5) Does the farmer agree to take part in the survey? was answered with yes.

[a10] [select_one]
76) When spraying, pesticide droplets are too tiny to drift and contaminate a nearby water point
Cheat sheet 5 - Use 5 dots scale. Read out all options.

[1]	agree not at all
[2]	agree a little
[3]	somewhat agree
[4]	rather agree
[5]	strongly agree
[98]	no response given

• Relevant when:
5) Does the farmer agree to take part in the survey? was answered with yes.

[a11] [select_one]

77) If there are many pests in the field then one should make the spraying mixture stronger

Cheat sheet 5 - Use 5 dots scale. Read out all options.

[1]	agree not at all
[2]	agree a little
[3]	somewhat agree
[4]	rather agree
[5]	strongly agree
[98]	no response given

• Relevant when:
5) Does the farmer agree to take part in the survey? was answered with yes.

[a12] [select_one]

78) It is not of my concern how the empty pesticide containers are discharged

Cheat sheet 5 - Use 5 dots scale. Read out all options.

[1]	agree not at all
[2]	agree a little
[3]	somewhat agree
[4]	rather agree
[5]	strongly agree
[98]	no response given

• Relevant when:
5) Does the farmer agree to take part in the survey? was answered with yes.

[a13] [select_one]

79) Testing one’s knapsack sprayer with water before mixing pesticides can save him/her from getting exposed while spraying

Cheat sheet 5 - Use 5 dots scale. Read out all options.

[1]	agree not at all
[2]	agree a little
[3]	somewhat agree
[4]	rather agree
[5]	strongly agree
[98]	no response given

• Relevant when:
5) Does the farmer agree to take part in the survey? was answered with yes.

[a14] [select_one]

80) Personal protective clothing should only be worn when it is not too hot

Cheat sheet 5 - Use 5 dots scale. Read out all options.

[1]	agree not at all
[2]	agree a little
[3]	somewhat agree
[4]	rather agree
[5]	strongly agree
[98]	no response given

• Relevant when:
5) Does the farmer agree to take part in the survey? was answered with yes.

[a15] [select_one]
81) Having separate spraying equipment for crops and animals is a wastage of money
Cheat sheet 5 - Use 5 dots scale. Read out all options.

[1]	agree not at all
[2]	agree a little
[3]	somewhat agree
[4]	rather agree
[5]	strongly agree
[98]	no response given

• Relevant when:
5) Does the farmer agree to take part in the survey? was answered with yes.

[a16] [select_one]
82) It is unnecessary to have more than one nozzle type for the same spraying equipment
Cheat sheet 5 - Use 5 dots scale. Read out all options.

[1]	agree not at all
[2]	agree a little
[3]	somewhat agree
[4]	rather agree
[5]	strongly agree
[98]	no response given

• Relevant when:
5) Does the farmer agree to take part in the survey? was answered with yes.

[ ] [note]
In the following I will aim to assess how often do you peform different tasks in relation to pesticide use in the past 12 months.

You can use the same dots as before but this time it indicates the frequency.

When you never do this activity, it is the small, when you rarely do this activity it is the second, when you sometimes do it, it is the third dot, when you often do it it is the foruth one and when you always do it you choose the biggest one.
explain 5 dote scale for frequency

• Relevant when:
5) Does the farmer agree to take part in the survey? was answered with yes.

[example_frequency] [select_one]

For example, please indicate, how often you take the bodaboda to go from your home to your farm in last year?

Cheat sheet 5 - Use 5 dots scale. Read out all options.

[5]	Never (0%)
[4]	Rarely (25%)
[3]	Sometimes (50%)
[2]	Often (75%)
[1]	Always (100%)
[98]	Don't want to answer *do not read out this to the farmer, only use it if farmer does not want to give an answer

• Relevant when:
5) Does the farmer agree to take part in the survey? was answered with yes.

[p1] [select_one]

83) I look for the name of the active ingredients when buying or using a given pesticide

Cheat sheet 5 - Use 5 dots scale. Read out all options.

[5]	Never (0%)
[4]	Rarely (25%)
[3]	Sometimes (50%)
[2]	Often (75%)
[1]	Always (100%)
[98]	Don't want to answer *do not read out this to the farmer, only use it if farmer does not want to give an answer

• Relevant when:
5) Does the farmer agree to take part in the survey? was answered with yes.

[p2] [select_one]
84) When buying and before using a new pesticide I read (or ask someone to read for me) the instructions on the label
Cheat sheet 5 - Use 5 dots scale. Read out all options.

[5]	Never (0%)
[4]	Rarely (25%)
[3]	Sometimes (50%)
[2]	Often (75%)
[1]	Always (100%)
[98]	Don't want to answer *do not read out this to the farmer, only use it if farmer does not want to give an answer

• Relevant when:
5) Does the farmer agree to take part in the survey? was answered with yes.

[p3] [select_one]
85) I mix stronger doses of the pesticides that I use on my crops to make sure it works
Cheat sheet 5 - Use 5 dots scale. Read out all options.

[5]	Never (0%)
[4]	Rarely (25%)
[3]	Sometimes (50%)
[2]	Often (75%)
[1]	Always (100%)
[98]	Don't want to answer *do not read out this to the farmer, only use it if farmer does not want to give an answer

• Relevant when:
5) Does the farmer agree to take part in the survey? was answered with yes.

[p4] [select_one]
86) I change/alternate the pesticides I use based on different modes of action
Cheat sheet 5 - Use 5 dots scale. Read out all options.

[5]	Never (0%)
[4]	Rarely (25%)
[3]	Sometimes (50%)
[2]	Often (75%)
[1]	Always (100%)
[98]	Don't want to answer *do not read out this to the farmer, only use it if farmer does not want to give an answer

• Relevant when:
5) Does the farmer agree to take part in the survey? was answered with yes.

[p5] [select_one]

87) In my home, we reuse empty pesticide containers for packing things such as sugar, salt, paraffin and others

Cheat sheet 5 - Use 5 dots scale. Read out all options.

[5]	Never (0%)
[4]	Rarely (25%)
[3]	Sometimes (50%)
[2]	Often (75%)
[1]	Always (100%)
[98]	Don't want to answer *do not read out this to the farmer, only use it if farmer does not want to give an answer

• Relevant when:
5) Does the farmer agree to take part in the survey? was answered with yes.

[p6] [select_one]
88) I look at the colour codes on a pesticide label to tell the hazard level of that pesticide
Cheat sheet 5 - Use 5 dots scale. Read out all options.

[5]	Never (0%)
[4]	Rarely (25%)
[3]	Sometimes (50%)
[2]	Often (75%)
[1]	Always (100%)
[98]	Don't want to answer *do not read out this to the farmer, only use it if farmer does not want to give an answer

• Relevant when:
5) Does the farmer agree to take part in the survey? was answered with yes.

[p7] [select_one]

89) In my home, I don’t allow my children to handle pesticides

Cheat sheet 5 - Use 5 dots scale. Read out all options.

[5]	Never (0%)
[4]	Rarely (25%)
[3]	Sometimes (50%)
[2]	Often (75%)
[1]	Always (100%)
[98]	Don't want to answer *do not read out this to the farmer, only use it if farmer does not want to give an answer

• Relevant when:
5) Does the farmer agree to take part in the survey? was answered with yes.

[p8] [select_one]

90) I do some other tasks in my field immediately or a few hours after spraying it with pesticides

Cheat sheet 5 - Use 5 dots scale. Read out all options.

[5]	Never (0%)
[4]	Rarely (25%)
[3]	Sometimes (50%)
[2]	Often (75%)
[1]	Always (100%)
[98]	Don't want to answer *do not read out this to the farmer, only use it if farmer does not want to give an answer

• Relevant when:
5) Does the farmer agree to take part in the survey? was answered with yes.

[p9] [select_one]

91) I only spray when it’s not windy to avoid pesticides drifting into the air

Cheat sheet 5 - Use 5 dots scale. Read out all options.

[5]	Never (0%)
[4]	Rarely (25%)
[3]	Sometimes (50%)
[2]	Often (75%)
[1]	Always (100%)
[98]	Don't want to answer *do not read out this to the farmer, only use it if farmer does not want to give an answer

• Relevant when:
5) Does the farmer agree to take part in the survey? was answered with yes.

[p10] [select_one]
92) I apply pesticides within less than 5m to nearby water ways/sources
Cheat sheet 5 - Use 5 dots scale. Read out all options.

[5]	Never (0%)
[4]	Rarely (25%)
[3]	Sometimes (50%)
[2]	Often (75%)
[1]	Always (100%)
[98]	Don't want to answer *do not read out this to the farmer, only use it if farmer does not want to give an answer

• Relevant when:
5) Does the farmer agree to take part in the survey? was answered with yes.

[p11] [select_one]
93) I mix strong concentrations so that I can kill all the insects in my field
Cheat sheet 5 - Use 5 dots scale. Read out all options.

[5]	Never (0%)
[4]	Rarely (25%)
[3]	Sometimes (50%)
[2]	Often (75%)
[1]	Always (100%)
[98]	Don't want to answer *do not read out this to the farmer, only use it if farmer does not want to give an answer

• Relevant when:
5) Does the farmer agree to take part in the survey? was answered with yes.

[p12] [select_one]

94) I leave my empty pesticide containers in the field or burn them

Cheat sheet 5 - Use 5 dots scale. Read out all options.

[5]	Never (0%)
[4]	Rarely (25%)
[3]	Sometimes (50%)
[2]	Often (75%)
[1]	Always (100%)
[98]	Don't want to answer *do not read out this to the farmer, only use it if farmer does not want to give an answer

• Relevant when:
5) Does the farmer agree to take part in the survey? was answered with yes.

[p13] [select_one]
95) I test my spraying equipment with water before the pesticide application
Cheat sheet 5 - Use 5 dots scale. Read out all options.

[5]	Never (0%)
[4]	Rarely (25%)
[3]	Sometimes (50%)
[2]	Often (75%)
[1]	Always (100%)
[98]	Don't want to answer *do not read out this to the farmer, only use it if farmer does not want to give an answer

• Relevant when:
5) Does the farmer agree to take part in the survey? was answered with yes.

[p14] [select_one]

96) I mix and spray pesticides in my field independent of the weather condition

Cheat sheet 5 - Use 5 dots scale. Read out all options.

[5]	Never (0%)
[4]	Rarely (25%)
[3]	Sometimes (50%)
[2]	Often (75%)
[1]	Always (100%)
[98]	Don't want to answer *do not read out this to the farmer, only use it if farmer does not want to give an answer

• Relevant when:
5) Does the farmer agree to take part in the survey? was answered with yes.

[p15] [select_one]

97) I use the same spraying equipment for my crops and animals

Cheat sheet 5 - Use 5 dots scale. Read out all options.

[5]	Never (0%)
[4]	Rarely (25%)
[3]	Sometimes (50%)
[2]	Often (75%)
[1]	Always (100%)
[98]	Don't want to answer *do not read out this to the farmer, only use it if farmer does not want to give an answer

• Relevant when:
5) Does the farmer agree to take part in the survey? was answered with yes.

[p16] [select_one]
98) With my spraying equipment, I use different nozzles, depending on what I am spraying against
Cheat sheet 5 - Use 5 dots scale. Read out all options.

[5]	Never (0%)
[4]	Rarely (25%)
[3]	Sometimes (50%)
[2]	Often (75%)
[1]	Always (100%)
[98]	Don't want to answer *do not read out this to the farmer, only use it if farmer does not want to give an answer

• Relevant when:
5) Does the farmer agree to take part in the survey? was answered with yes.

[ ] [note]
FORMER beh QUESTIONNAIRE STARTS

[ ] [note]

#E. Personal Protective Equipment

• Relevant when:

5) Does the farmer agree to take part in the survey? was answered with yes.

[time_e] [time]

Please take the time

• Relevant when:

5) Does the farmer agree to take part in the survey? was answered with yes.

E. Personal Protective Equipment

[ ] [note]

100) [Show the participant the guide for personal protective equipment]
On this sheet you can see the different protective equipment used to prepare ar apply pesticides. Do you have access or any of this equipment in your household?

Refer to Cheat sheet 6 "PPE"

[ppe01a] [select_one]
100.1) Gumboots

[0]	No
[1]	Yes
[98]	Don't know

[ppe02a] [select_one]
100.2) Closed shoes

[0]	No
[1]	Yes
[98]	Don't know

[ppe03a] [select_one]
100.3) Long pants

[0]	No
[1]	Yes
[98]	Don't know

[ppe04a] [select_one]

100.4) Waterproof pants

[0]	No

[1]	Yes
[98]	Don't know

[ppe05a] [select_one]
100.5) Long sleeved shirt or blouse which covers arms

[0]	No
[1]	Yes
[98]	Don't know

[ppe06a] [select_one]
100.6) Rubber apron

[0]	No
[1]	Yes
[98]	Don't know

[ppe07a] [select_one]
100.7) Poncho or overcoat / raincoat

[0]	No
[1]	Yes
[98]	Don't know

[ppe08a] [select_one]
100.8) Overall or Kimono

[0]	No
[1]	Yes
[98]	Don't know

[ppe09a] [select_one]
100.9) Mask without carbon filter (same or similar mask as used to protect against covid)

[0]	No
[1]	Yes
[98]	Don't know

• Relevant when:
100.9) Mask without carbon filter (same or similar mask as used to protect against covid) was answered with Yes.

[ppe09_covid] [select_one]
101.9a) Mask without carbon filter - Did you get access to the mask because of covid?

[0]	No
[1]	Yes
[98]	Don't know

• Relevant when:
100.9) Mask without carbon filter (same or similar mask as used to protect against covid) was answered with Yes.

[ppe09_covid_access] [text]
101.9a) Mask without carbon filter - who providied you the mask to protect against covid?

[ppe10a] [select_one]
100.10) Mask with carbon filter

[0]	No
[1]	Yes
[98]	Don't know

[ppe11a] [select_one]
100.11) Gloves - one way

[0]	No
[1]	Yes
[98]	Don't know

[ppe15a] [select_one]
100.12) Gloves - Chemical resistant

[0]	No
[1]	Yes
[98]	Don't know

[ppe16a] [select_one]
100.13) Gloves - woven

[0]	No
[1]	Yes
[98]	Don't know

[ppe13a] [select_one]
100.14) Glasses

[0]	No
[1]	Yes
[98]	Don't know

[ppe12a] [select_one]

100.15) Cap

[0]	No

[1]	Yes
[98]	Don't know

[ppe14a] [select_one]

100.16) any other equipment?

[0]	No
[1]	Yes
[98]	Don't know

• Relevant when:

5) Does the farmer agree to take part in the survey? was answered with yes.

[ ] [note]

Now we would like to know how often you used the PPE you have at your home in the past 12 month. First, for work in the field in general, for example for mentaining the filed or planting or harvesting crops but not for pesticide use. And second how often you use it when applying pesticices in the past 12 months.

You can use the same dots as before to indicate the frequency.

When you never use the PPE, it is the small, when you rarely use the PPE (about 25% of your timeit is the second, when you sometimes use it (about 50% of your time), it is the third dot, when you often use it (75% of your timeit is the foruth one and when you always use it you choose the biggest one.

Refer to Cheat sheet 6 "PPE"

• Relevant when:
100.16) any other equipment? was answered with Yes.

[ppe14a_sp] [text]
101.16a) What is that other equipment?

• Relevant when:
100.16) any other equipment? was answered with Yes.

[ppe14b] [select_one]
101.16b) How often do you use this protective equipment when working in the field?
Cheat sheet 5 - Use 5 dots scale. Read out all options.

[5]	Never (0%)
[4]	Rarely (25%)
[3]	Sometimes (50%)
[2]	Often (75%)
[1]	Always (100%)
[98]	Don't want to answer *do not read out this to the farmer, only use it if farmer does not want to give an answer

• Relevant when:
100.16) any other equipment? was answered with Yes.

[ppe14c] [select_one]

101.16c) How often do you use this protective equipment when preparing or applying pesticides?

Cheat sheet 5 - Use 5 dots scale. Read out all options.

[5]	Never (0%)
[4]	Rarely (25%)
[3]	Sometimes (50%)
[2]	Often (75%)
[1]	Always (100%)
[98]	Don't want to answer *do not read out this to the farmer, only use it if farmer does not want to give an answer

• Relevant when:
100.1) Gumboots was answered with Yes.

[ppe01b] [select_one]
101.1a) Gumboots - How often do you use this protective equipment when working in the field??
Cheat sheet 5 - Use 5 dots scale. Read out all options.

[5]	Never (0%)
[4]	Rarely (25%)
[3]	Sometimes (50%)
[2]	Often (75%)
[1]	Always (100%)
[98]	Don't want to answer *do not read out this to the farmer, only use it if farmer does not want to give an answer

• Relevant when:
100.1) Gumboots was answered with Yes.

[ppe01c] [select_one]

101.1b) Gumboots - How often do you use this protective equipment when preparing or applying pesticides?

Cheat sheet 5 - Use 5 dots scale. Read out all options.

[5]	Never (0%)
[4]	Rarely (25%)
[3]	Sometimes (50%)
[2]	Often (75%)
[1]	Always (100%)
[98]	Don't want to answer *do not read out this to the farmer, only use it if farmer does not want to give an answer

• Relevant when:
100.2) Closed shoes was answered with Yes.

[ppe02b] [select_one]
101.2a) closed shoes - How often do you use this protective equipment when working in the field?
Cheat sheet 5 - Use 5 dots scale. Read out all options.

[5]	Never (0%)
[4]	Rarely (25%)
[3]	Sometimes (50%)
[2]	Often (75%)
[1]	Always (100%)
[98]	Don't want to answer *do not read out this to the farmer, only use it if farmer does not want to give an answer

• Relevant when:
100.2) Closed shoes was answered with Yes.

[ppe02c] [select_one]
101.2b) closed shoes - How often do you use this protective equipment when preparing or applying pesticides?
Cheat sheet 5 - Use 5 dots scale. Read out all options.

[5]	Never (0%)
[4]	Rarely (25%)
[3]	Sometimes (50%)
[2]	Often (75%)
[1]	Always (100%)
[98]	Don't want to answer *do not read out this to the farmer, only use it if farmer does not want to give an answer

• Relevant when:
100.3) Long pants was answered with Yes.

[ppe03b] [select_one]

101.3a) Long pants - How often do you use this protective equipment when working in the field?

Cheat sheet 5 - Use 5 dots scale. Read out all options.

[5]	Never (0%)
[4]	Rarely (25%)
[3]	Sometimes (50%)
[2]	Often (75%)
[1]	Always (100%)
[98]	Don't want to answer *do not read out this to the farmer, only use it if farmer does not want to give an answer

• Relevant when:
100.3) Long pants was answered with Yes.

[ppe03c] [select_one]
101.3b) Long pants - How often do you use this protective equipment when preparing or applying pesticides?
Cheat sheet 5 - Use 5 dots scale. Read out all options.

[5]	Never (0%)
[4]	Rarely (25%)
[3]	Sometimes (50%)
[2]	Often (75%)
[1]	Always (100%)
[98]	Don't want to answer *do not read out this to the farmer, only use it if farmer does not want to give an answer

• Relevant when:
100.4) Waterproof pants was answered with Yes.

[ppe04b] [select_one]

101.4a) Waterproof pants - How often do you use this protective equipment when working in the field?

Cheat sheet 5 - Use 5 dots scale. Read out all options.

[5]	Never (0%)
[4]	Rarely (25%)
[3]	Sometimes (50%)
[2]	Often (75%)
[1]	Always (100%)
[98]	Don't want to answer *do not read out this to the farmer, only use it if farmer does not want to give an answer

• Relevant when:
100.4) Waterproof pants was answered with Yes.

[ppe04c] [select_one]

101.4b) Waterproof pants - How often do you use this protective equipment when preparing or applying pesticides?

Cheat sheet 5 - Use 5 dots scale. Read out all options.

[5]	Never (0%)
[4]	Rarely (25%)
[3]	Sometimes (50%)
[2]	Often (75%)
[1]	Always (100%)
[98]	Don't want to answer *do not read out this to the farmer, only use it if farmer does not want to give an answer

• Relevant when:
100.5) Long sleeved shirt or blouse which covers arms was answered with Yes.

[ppe05b] [select_one]

101.5a) Long sleeved shirt or blouse which covers arms - How often do you use this protective equipment when working in the field?

Cheat sheet 5 - Use 5 dots scale. Read out all options.

[5]	Never (0%)
[4]	Rarely (25%)
[3]	Sometimes (50%)
[2]	Often (75%)
[1]	Always (100%)
[98]	Don't want to answer *do not read out this to the farmer, only use it if farmer does not want to give an answer

• Relevant when:
100.5) Long sleeved shirt or blouse which covers arms was answered with Yes.

[ppe05c] [select_one]

101.5b) Long sleeved shirt or blouse which covers arms - How often do you use this protective equipment when preparing or applying pesticides?

Cheat sheet 5 - Use 5 dots scale. Read out all options.

[5]	Never (0%)
[4]	Rarely (25%)
[3]	Sometimes (50%)
[2]	Often (75%)
[1]	Always (100%)
[98]	Don't want to answer *do not read out this to the farmer, only use it if farmer does not want to give an answer

• Relevant when:
100.6) Rubber apron was answered with Yes.

[ppe06b] [select_one]

101.6a) Rubber apron - How often do you use this protective equipment when working in the field?

Cheat sheet 5 - Use 5 dots scale. Read out all options.

[5]	Never (0%)
[4]	Rarely (25%)
[3]	Sometimes (50%)
[2]	Often (75%)
[1]	Always (100%)
[98]	Don't want to answer *do not read out this to the farmer, only use it if farmer does not want to give an answer

• Relevant when:
100.6) Rubber apron was answered with Yes.

[ppe06c] [select_one]
101.6b) Rubber apron - How often do you use this protective equipment when preparing or applying pesticides?
Cheat sheet 5 - Use 5 dots scale. Read out all options.

[5]	Never (0%)
[4]	Rarely (25%)
[3]	Sometimes (50%)
[2]	Often (75%)
[1]	Always (100%)
[98]	Don't want to answer *do not read out this to the farmer, only use it if farmer does not want to give an answer

• Relevant when:
100.7) Poncho or overcoat / raincoat was answered with Yes.

[ppe07b] [select_one]

101.7a) Poncho or overcoat / raincoat - How often do you use this protective equipment when working in the field?

Cheat sheet 5 - Use 5 dots scale. Read out all options.

[5]	Never (0%)
[4]	Rarely (25%)
[3]	Sometimes (50%)
[2]	Often (75%)
[1]	Always (100%)
[98]	Don't want to answer *do not read out this to the farmer, only use it if farmer does not want to give an answer

• Relevant when:
100.7) Poncho or overcoat / raincoat was answered with Yes.

[ppe07c] [select_one]

101.7b) Poncho or overcoat / raincoat - How often do you use this protective equipment when preparing or applying pesticides?

Cheat sheet 5 - Use 5 dots scale. Read out all options.

[5]	Never (0%)
[4]	Rarely (25%)
[3]	Sometimes (50%)
[2]	Often (75%)
[1]	Always (100%)
[98]	Don't want to answer *do not read out this to the farmer, only use it if farmer does not want to give an answer

• Relevant when:
100.8) Overall or Kimono was answered with Yes.

[ppe08b] [select_one]

101.8a) Overall or kimono - How often do you use this protective equipment when working in the field?

Cheat sheet 5 - Use 5 dots scale. Read out all options.

[5]	Never (0%)
[4]	Rarely (25%)
[3]	Sometimes (50%)
[2]	Often (75%)
[1]	Always (100%)
[98]	Don't want to answer *do not read out this to the farmer, only use it if farmer does not want to give an answer

• Relevant when:
100.8) Overall or Kimono was answered with Yes.

[ppe08c] [select_one]

101.8b) Overall or kimono - How often do you use this protective equipment when preparing or applying pesticides?

Cheat sheet 5 - Use 5 dots scale. Read out all options.

[5]	Never (0%)
[4]	Rarely (25%)
[3]	Sometimes (50%)
[2]	Often (75%)
[1]	Always (100%)
[98]	Don't want to answer *do not read out this to the farmer, only use it if farmer does not want to give an answer

• Relevant when:
100.9) Mask without carbon filter (same or similar mask as used to protect against covid) was answered with Yes.

[ppe09_covid_fre] [select_one]

101.9a) Mask without carbon filter - How often do you use this protective equipment in the public to protect your self against covid?

Cheat sheet 5 - Use 5 dots scale. Read out all options.

[5]	Never (0%)
[4]	Rarely (25%)
[3]	Sometimes (50%)
[2]	Often (75%)
[1]	Always (100%)
[98]	Don't want to answer *do not read out this to the farmer, only use it if farmer does not want to give an answer

• Relevant when:
100.9) Mask without carbon filter (same or similar mask as used to protect against covid) was answered with Yes.

[ppe09b] [select_one]
101.9b) Mask without carbon filter - How often do you use this protective equipment when working in the field?
Cheat sheet 5 - Use 5 dots scale. Read out all options.

[5]	Never (0%)
[4]	Rarely (25%)
[3]	Sometimes (50%)
[2]	Often (75%)
[1]	Always (100%)
[98]	Don't want to answer *do not read out this to the farmer, only use it if farmer does not want to give an answer

• Relevant when:
100.9) Mask without carbon filter (same or similar mask as used to protect against covid) was answered with Yes.

[ppe09c] [select_one]

101.9c) Mask without carbon filter - How often do you use this protective equipment when preparing or applying pesticides?

Cheat sheet 5 - Use 5 dots scale. Read out all options.

[5]	Never (0%)
[4]	Rarely (25%)
[3]	Sometimes (50%)
[2]	Often (75%)
[1]	Always (100%)
[98]	Don't want to answer *do not read out this to the farmer, only use it if farmer does not want to give an answer

• Relevant when:
100.9) Mask without carbon filter (same or similar mask as used to protect against covid) was answered with Yes.

[ppe09_repalce] [select_one]
101.9d) Mask without carbon filter - How often do repalce the mask?

[5]	Never
[4]	Up to six times since the last visit
[3]	Approximately every month
[2]	Approximately every week
[1]	After every use
[98]	Don't want to answer *do not read out this to the farmer, only use it if farmer does not want to give an answer

• Relevant when:
100.10) Mask with carbon filter was answered with Yes.

[ppe10b] [select_one]

101.10a) Mask with carbon filter - How often do you use this protective equipment when working in the field?

Cheat sheet 5 - Use 5 dots scale. Read out all options.

[5]	Never (0%)
[4]	Rarely (25%)
[3]	Sometimes (50%)
[2]	Often (75%)
[1]	Always (100%)
[98]	Don't want to answer *do not read out this to the farmer, only use it if farmer does not want to give an answer

• Relevant when:
100.10) Mask with carbon filter was answered with Yes.

[ppe10c] [select_one]

101.10b) Mask with carbon filter - How often do you use this protective equipment when preparing or applying pesticides?

Cheat sheet 5 - Use 5 dots scale. Read out all options.

[5]	Never (0%)
[4]	Rarely (25%)
[3]	Sometimes (50%)
[2]	Often (75%)
[1]	Always (100%)
[98]	Don't want to answer *do not read out this to the farmer, only use it if farmer does not want to give an answer

• Relevant when:
100.11) Gloves - one way was answered with Yes.

[ppe11b] [select_one]

101.11a) Gloves -one way - How often do you use this protective equipment when working in the field?

Cheat sheet 5 - Use 5 dots scale. Read out all options.

[5]	Never (0%)
[4]	Rarely (25%)
[3]	Sometimes (50%)
[2]	Often (75%)
[1]	Always (100%)
[98]	Don't want to answer *do not read out this to the farmer, only use it if farmer does not want to give an answer

• Relevant when:
100.11) Gloves - one way was answered with Yes.

[ppe11c] [select_one]

101.11b) Gloves -one way - How often do you use this protective equipment when preparing or applying pesticides?

Cheat sheet 5 - Use 5 dots scale. Read out all options.

[5]	Never (0%)
[4]	Rarely (25%)
[3]	Sometimes (50%)
[2]	Often (75%)
[1]	Always (100%)
[98]	Don't want to answer *do not read out this to the farmer, only use it if farmer does not want to give an answer

• Relevant when:
100.11) Gloves - one way was answered with Yes.

[ppe11c_repalce] [select_one]
101.9c) Gloves -one way - How often do repalce the gloves?

[5]	Never
[4]	Up to six times since the last visit
[3]	Approximately every month
[2]	Approximately every week
[1]	After every use
[98]	Don't want to answer *do not read out this to the farmer, only use it if farmer does not want to give an answer

• Relevant when:
100.12) Gloves - Chemical resistant was answered with Yes.

[ppe15b] [select_one]

101.12a) Gloves - chemical resitant- How often do you use this protective equipment when preparing or applying pesticides?

Cheat sheet 5 - Use 5 dots scale. Read out all options.

[5]	Never (0%)
[4]	Rarely (25%)
[3]	Sometimes (50%)
[2]	Often (75%)
[1]	Always (100%)
[98]	Don't want to answer *do not read out this to the farmer, only use it if farmer does not want to give an answer

• Relevant when:
100.12) Gloves - Chemical resistant was answered with Yes.

[ppe15c] [select_one]

101.12b) Gloves - chemical resitant - How often do you use this protective equipment when working in the field?

Cheat sheet 5 - Use 5 dots scale. Read out all options.

[5]	Never (0%)
[4]	Rarely (25%)
[3]	Sometimes (50%)
[2]	Often (75%)
[1]	Always (100%)
[98]	Don't want to answer *do not read out this to the farmer, only use it if farmer does not want to give an answer

• Relevant when:
100.13) Gloves - woven was answered with Yes.

[ppe16b] [select_one]

101.13a) Gloves - woven- How often do you use this protective equipment when preparing or applying pesticides?

Cheat sheet 5 - Use 5 dots scale. Read out all options.

[5]	Never (0%)
[4]	Rarely (25%)
[3]	Sometimes (50%)
[2]	Often (75%)
[1]	Always (100%)
[98]	Don't want to answer *do not read out this to the farmer, only use it if farmer does not want to give an answer

• Relevant when:
100.13) Gloves - woven was answered with Yes.

[ppe16c] [select_one]

101.13b) Gloves - woven - How often do you use this protective equipment when working in the field?

Cheat sheet 5 - Use 5 dots scale. Read out all options.

[5]	Never (0%)
[4]	Rarely (25%)
[3]	Sometimes (50%)
[2]	Often (75%)
[1]	Always (100%)
[98]	Don't want to answer *do not read out this to the farmer, only use it if farmer does not want to give an answer

• Relevant when:
100.14) Glasses was answered with Yes.

[ppe13b] [select_one]
101.14a) Glasses - How often do you use this protective equipment when working in the field?
Cheat sheet 5 - Use 5 dots scale. Read out all options.

[5]	Never (0%)
[4]	Rarely (25%)
[3]	Sometimes (50%)
[2]	Often (75%)
[1]	Always (100%)
[98]	Don't want to answer *do not read out this to the farmer, only use it if farmer does not want to give an answer

• Relevant when:
100.14) Glasses was answered with Yes.

[ppe13c] [select_one]
101.14b) Glasses - How often do you use this protective equipment when preparing or applying pesticides?
Cheat sheet 5 - Use 5 dots scale. Read out all options.

[5]	Never (0%)
[4]	Rarely (25%)
[3]	Sometimes (50%)
[2]	Often (75%)
[1]	Always (100%)
[98]	Don't want to answer *do not read out this to the farmer, only use it if farmer does not want to give an answer

• Relevant when:
100.15) Cap was answered with Yes.

[ppe12b] [select_one]

101.15a) Cap - How often do you use this protective equipment when working in the field?

Cheat sheet 5 - Use 5 dots scale. Read out all options.

[5]	Never (0%)
[4]	Rarely (25%)
[3]	Sometimes (50%)
[2]	Often (75%)
[1]	Always (100%)
[98]	Don't want to answer *do not read out this to the farmer, only use it if farmer does not want to give an answer

• Relevant when:
100.15) Cap was answered with Yes.

[ppe12c] [select_one]
101.15b) Cap - How often do you use this protective equipment when preparing or applying pesticides?
Cheat sheet 5 - Use 5 dots scale. Read out all options.

[5]	Never (0%)
[4]	Rarely (25%)
[3]	Sometimes (50%)
[2]	Often (75%)
[1]	Always (100%)
[98]	Don't want to answer *do not read out this to the farmer, only use it if farmer does not want to give an answer

• Relevant when:
31) Do you apply or mix pesticides when you are being pregnant? was answered with Yes.

[ppe_preg2] [select_one]
102) Do you change anything regarding the use of any PPE during pesticide application when you are pregnant?

[1]	yes
[0]	no

• Relevant when:
102) Do you change anything regarding the use of any PPE during pesticide application when you are pregnant? was answered with yes.

[ppe_preg3] [text]
102.1) What do you change?

• Relevant when:
31) Do you apply or mix pesticides when you are being pregnant? was answered with Yes.

[ppe_preg4] [select_one]
102.2) How frequently do you wear PPE during pesticide application when you are pregnant?

[1]	Never (0%)
[2]	Rarely (25%)
[3]	Sometimes (50%)
[4]	Often (75%)
[5]	Always (100%)
[98]	no response given

• Relevant when:
5) Does the farmer agree to take part in the survey? was answered with yes.

[time_ranas1] [time]
Please take the time

• Relevant when:
5) Does the farmer agree to take part in the survey? was answered with yes.

[ ] [note]
Now, please think about when you are about to go spraying and you are preparing for handling your pesticides….

• Relevant when:
5) Does the farmer agree to take part in the survey? was answered with yes.

[ ] [note]
In the following I will ask you about the use of this items

• Relevant when:
5) Does the farmer agree to take part in the survey? was answered with yes.

[hinder] [select_multiple]

103) Do you see any difficulties that might hinder you from wearing the PPE items you mentioned when handling pesticides?

Do not read out. Select all mentioned answers,

[1]	Financial constraints to buy
[2]	Not knowing where to buy
[3]	Sweating
[4]	Hamper from work
[5]	Other person may steal it
[6]	When being sick
[7]	PPE is not in a good condition/ broken
[8]	Can not think of any
[9]	Not owning PPEs
[10]	Lack of knowledge on PPEs
[11]	Being pregnant
[96]	Other
[98]	No response given

• Relevant when:

103) Do you see any difficulties that might hinder you from wearing the PPE items you mentioned when handling pesticides? was answered with Other.

[hinder_y] [text]

103.4) Specify other

• Relevant when:
5) Does the farmer agree to take part in the survey? was answered with yes.

[overcome] [select_multiple]
104) Do you have a plan how to overcome these difficulties?
Do not read out. Select all mentioned answers,

[1]	Buy them (one by one)
[2]	Keep them safe
[3]	Having more than one set
[4]	Ask someone where PPE are sold
[5]	Spray with PPE only when weather is cold
[6]	Being careful when using pesticides without PPE
[9]	Ask someone to borrow their PPE
[7]	NO PLAN
[10]	Getting medical treatment
[11]	Hiring labour
[12]	Use no PPE / not all PPE
[13]	Government / Organisations should improve access / sensitisation
[95]	No plan because no problem
[96]	Other
[98]	No response given

• Relevant when:
104) Do you have a plan how to overcome these difficulties? was answered with Other.

[overcome_y] [text]
104.1) Specify other

• Relevant when:
5) Does the farmer agree to take part in the survey? was answered with yes.

[commit] [select_one]
105) How strongly do you feel committed to wearing the PPE items you have when handling pesticides?
Cheat sheet 5 - Use 5 dots scale. Read out all options.

[1]	not at all committed
[2]	a little committed
[3]	somewhat committed
[4]	rather committed
[5]	very much committed
[98]	no response given

• Relevant when:
5) Does the farmer agree to take part in the survey? was answered with yes.

[ ] [note]
In the following interview part we will be asking you about your thoughts and opinions on the usage of all the recommended PPE this person is wearing [show picture]. (There are no right or wrong answers.)
When we speak about handling pesticides, we mean the following actions: mixing substances, preparing the application instruments, spraying in the fields, as well as cleaning up the equipment afterwards.
When we talk about PPE, we are considering all those items that can help you protect your health when handling different substances. This means covering all your body parts, including arms, legs, and feet, hand, eyes, mouth, and head.The person on this picture wears all the recommended PPE to cover all body parts.
Please also see this person on the picture as a reminder of what PPE items can be used to cover all body parts.Please remind yourself of this picture when answering the following questions.
Please explain the farmer the different set of PPE which are recommended and make sure the cheat sheet showing all recommended PPE is displayed during the whole interview! Cheat sheet 7 - recommended PPE

• Relevant when:
5) Does the farmer agree to take part in the survey? was answered with yes.

[ ] [note]
In the following I will aim to assess how much you agree with a feeling or statement. You can use these dots to indicate your opinion as before. When you agree not at all, it is the small, when you aree a little it is the second, when you somewhat agree it is the third dot, when you rather agree it is this dot and when you agree very much you choose this one, the big one.
explain 5 dote scale

• Relevant when:
5) Does the farmer agree to take part in the survey? was answered with yes.

agreement with statement

[intent] [select_one]

106) How strong is your intention to always wear all the recommended PPE when handling pesticides in the future?

Cheat sheet 5 - Use 5 dots scale. Read out all options.

[1]	no intention at all
[2]	intent a little
[3]	somewhat intend
[4]	rather intend
[5]	intent very much
[98]	no response given

[ppe3] [select_one]

107) To what extent do you think that wearing all the recommended PPE when handling pesticides can protect you from getting sick?

Cheat sheet 5 - Use 5 dots scale. Read out all options.

[1]	not at all
[2]	a little
[3]	somewhat/ to a medium extent
[4]	rather well / much
[5]	very much
[98]	no response given

• Relevant when:

5) Does the farmer agree to take part in the survey? was answered with yes.

PPE_attitudes

[whynoppe] [select_multiple]

108) What are the reasons you are not using some of the PPEs when handling pesticides?

[1]	Too expensive
[2]	Too uncomfortable
[3]	Not available
[4]	Wrong size
[5]	Uses all the PPE
[6]	Organic pesticides do not require PPE
[7]	Don't care about PPE
[8]	Not aware of PPE / Lack of knowledge
[9]	Not aware where to buy / prices
[10]	Broken PPE
[11]	Not aware of pesticide health risk
[12]	Clothes too nice
[13]	Stolen PPE
[14]	Sickness / Injury
[77]	Unclear reason
[96]	Other
[98]	No response given

• Relevant when:
108) What are the reasons you are not using some of the PPEs when handling pesticides? was answered with Other.

[whynoppe_sp] [text]
108.1) Specify other

[full_set] [select_one]
109) How expensive do you consider it to buy of all of the recommended PPE items?

[1]	not at all expensive
[2]	little expensive
[3]	somewhat expensive
[4]	rather expensive
[5]	very expensive
[98]	no response given

[where] [select_one]
110) Where would you go to buy all of this equipment?
Please rate the respondents answer accordingly.

[1]	participant knows where he can buy the equipment
[0]	participant does not know

[intent] [select_one]

111) How strong is your intention to buy all of the recommended PPE items for your personal use?

Cheat sheet 5 - Use 5 dots scale. Read out all options.

[1]	no intention at all
[2]	intent a little
[3]	somewhat intend
[4]	rather intend
[5]	intent very much
[98]	no response given

[fitting] [select_one]

112) How fitting are the recommended PPE for you (e.g. size, cut)?

Cheat sheet 5 - Use 5 dots scale. Read out all options.

[1]	not at all
[2]	a little
[3]	somewhat/ to a medium extent
[4]	rather well / much
[5]	very much
[98]	no response given

[time] [select_one]

113) How time-consuming do you think it is to put on all the recommended PPE when handling pesticides?

Cheat sheet 5 - Use 5 dots scale. Read out all options.

[1]	not at all
[2]	a little
[3]	somewhat/ to a medium extent
[4]	rather well / much
[5]	very much
[98]	no response given

[comf] [select_one]

114) To what extent do you feel it is uncomfortable to always wear all the recommended PPE when handling pesticides?

Cheat sheet 5 - Use 5 dots scale. Read out all options.

[1]	not at all uncomfortable
[2]	a little uncomfortable
[3]	somewhat uncomfortable
[4]	rather uncomfortable
[5]	very uncomfortable
[98]	no response given

[look] [select_one]

115) How proud would you feel if you were wearing all the recommended PPE when handling pesticides?

Cheat sheet 5 - Use 5 dots scale. Read out all options.

[1]	not at all
[2]	a little
[3]	somewhat/ to a medium extent
[4]	rather well / much
[5]	very much
[98]	no response given

[annoy] [select_one]

116) To what extent would others think you are weak when wearing all the recommended PPE?

Cheat sheet 5 - Use 5 dots scale. Read out all options.

[1]	not at all
[2]	a little
[3]	somewhat/ to a medium extent
[4]	rather well / much
[5]	very much
[98]	no response given

[glad] [select_one]

117) Now, considering the potential disadvantages of wearing all of the recommended PPE items against its advantages, how advantageous do you evaluate the usage of PPE as recommended?

Cheat sheet 5 - Use 5 dots scale. Read out all options.

[1]	not advantageous at all
[2]	a little advantageous
[3]	somewhat advantageous
[4]	rather advantageous
[5]	very advantegous
[98]	no response given

• Relevant when:

5) Does the farmer agree to take part in the survey? was answered with yes.

PPE_norms

[fam] [select_one]

118) How many farmers on neighboring farms are using all the recommended PPE when handling pestices?

Cheat sheet 5 - Use 5 dots scale. Read out all options.

[1]	(almost) nobody (0%)
[2]	some of them (25%)
[3]	half of them (50%)
[4]	most of them (75%)
[5]	(almost) everybody (100%)
[98]	no response given

• Relevant when:
6) Gender was answered with female.

[match1] [select_one]
119) What do you think, how many women in your neighbourhood are using all the recommended PPE when handling pesticides?
Cheat sheet 5 - Use 5 dots scale. Read out all options.

[1]	(almost) nobody (0%)
[2]	some of them (25%)
[3]	half of them (50%)
[4]	most of them (75%)
[5]	(almost) everybody (100%)
[98]	no response given

• Relevant when:
6) Gender was answered with male.

[match2] [select_one]
119) What do you think, how many men in your neighbourhood are using all the recommended PPE when handling pesticides?
Cheat sheet 5 - Use 5 dots scale. Read out all options.

[1]	(almost) nobody (0%)
[2]	some of them (25%)
[3]	half of them (50%)
[4]	most of them (75%)
[5]	(almost) everybody (100%)
[98]	no response given

[important] [select_one]

120) People who are important to you, how strongly do they think that you should be wearing all the recommended PPE when handling pesticides?

Cheat sheet 5 - Use 5 dots scale. Read out all options.

[1]	not at all
[2]	a little
[3]	somewhat/ to a medium extent
[4]	rather well / much
[5]	very much
[98]	no response given

[partner] [select_one]

121) To what extent do you think that your wife (or partner) wants you to wear all the recommended PPE when handling pesticides?

Cheat sheet 5 - Use 5 dots scale. Read out all options.

[1]	not at all
[2]	a little
[3]	somewhat/ to a medium extent
[4]	rather well / much
[5]	very much
[98]	no response given

[children] [select_one]

122) If you have any adult children, how strongly do you think they want you to protect yourself wearing all the recommended PPE when handling pesticides?

Cheat sheet 5 - Use 5 dots scale. Read out all options.

[1]	not at all
[2]	a little
[3]	somewhat
[4]	rather
[5]	very much
[77]	not applicable
[98]	no response given

[oblig] [select_one]

123) To what extent do you feel a personal obligation to wear the all the recommended PPE when handling pesticides?

Cheat sheet 5 - Use 5 dots scale. Read out all options.

[1]	not at all
[2]	a little
[3]	somewhat/ to a medium extent
[4]	rather well / much
[5]	very much
[98]	no response given

[you] [select_one]

124) How important is it for you to wear all the recommended PPE when handling pesticides?

Cheat sheet 5 - Use 5 dots scale. Read out all options.

[1]	not at all
[2]	a little
[3]	somewhat/ to a medium extent
[4]	rather well / much
[5]	very much
[98]	no response given

• Relevant when:

5) Does the farmer agree to take part in the survey? was answered with yes.

PPE_abilities

[ ] [note]

In the next section we would like to know how you estimate your own abilities in using PPE. There are no right or wrong answers, we are simply interested in what you think personally. Please answer as bluntly as possible.

[buy_all] [select_one]

125) How confident are you that you can buy all the recommended PPE when handling pesticides?

Cheat sheet 5 - Use 5 dots scale. Read out all options.

[1]	not at all confident
[2]	a little confident
[3]	somewhat confident
[4]	rather confident
[5]	very confident
[98]	no response given

[waer_all] [select_one]

126) How confident are you that you can remember to wear all the recommended PPE when handling pesticides?

Cheat sheet 5 - Use 5 dots scale. Read out all options.

[1]	not at all confident
[2]	a little confident
[3]	somewhat confident
[4]	rather confident
[5]	very confident
[98]	no response given

[next_m] [select_one]

127) How confident are you that you will always wear all the recommended PPE when handling pesticides even when it is very hot?

Cheat sheet 5 - Use 5 dots scale. Read out all options.

[1]	not at all confident
[2]	a little confident
[3]	somewhat confident
[4]	rather confident
[5]	very confident
[98]	no response given

[access] [select_one]

128) How confident are you that you would be able to start wearing all the recommended PPE again even if you did not do so for a while (e.g. when it was broken and needed replacement)?

Cheat sheet 5 - Use 5 dots scale. Read out all options.

[1]	not at all confident
[2]	a little confident
[3]	somewhat confident
[4]	rather confident
[5]	very confident
[98]	no response given

• Relevant when:
5) Does the farmer agree to take part in the survey? was answered with yes.

PPE_self-reg

[plan_1] [select_multiple]

129) Do you have a plan how to buy all the PPE to cover all body parts [indicate picture again]?

Do not read out. Select all mentioned answers,

[1]	Buy them one by one
[2]	Ask someone where PPE are sold
[3]	Save money to buy them
[4]	Selling specific items to buy them
[5]	NO PLAN
[6]	Does not care
[7]	Seek free PPE
[8]	Take a loan to buy PPE
[9]	Improve local access
[96]	Other
[98]	No response given

• Relevant when:
129) Do you have a plan how to buy all the PPE to cover all body parts [indicate picture again]? was answered with Other.

[plan_1_sp] [text]
129.1) Specify other

[plan_2] [select_multiple]
130) Imagine you had all the PPE, do you have a plan how to make sure you always wear them?
Do not read out. Select all mentioned answers,

[1]	Wash them regulary
[2]	Having more than one set
[3]	Putting them in a place to remember wearing them
[4]	NO PLAN
[5]	Does not care
[6]	Health cautiousness
[7]	Set alarm / Someone to remind me
[8]	Keep them with care
[9]	Putting under sunshine after spraying
[96]	Other
[98]	No response given

• Relevant when:
130) Imagine you had all the PPE, do you have a plan how to make sure you always wear them? was answered with Other.

[plan_2_sp] [text]
131.1) Specify other

[effort] [select_one]
132) To what extent do you make a conscious effort to always wear all the recommended PPE?
Cheat sheet 5 - Use 5 dots scale. Read out all options.

[1]	not at all
[2]	a little
[3]	somewhat/ to a medium extent
[4]	rather well / much
[5]	very much
[98]	no response given

• Relevant when:
5) Does the farmer agree to take part in the survey? was answered with yes.

[ ] [note]
In the following, please rate your personal risk in relation to pesticide usage depending on given circumstances.

• Relevant when:
5) Does the farmer agree to take part in the survey? was answered with yes.

Rate risk in relation to pesticide usage

[no_ppe] [select_one]

133) How dangerous do you think is it for your health when you are handling pesticides without all the recommended PPE?

Cheat sheet 5 - Use 5 dots scale. Read out all options.

[1]	not at all dangerous
[2]	a little dangerous
[3]	somewhat dangerous
[4]	rather dangerous
[5]	very dangerous
[98]	no response given

[symp_no_ppe] [select_one]

134) How likely do you think it is that you suffer from acute symptoms from using pesticides without wearing all the recommended PPE?

Cheat sheet 5 - Use 5 dots scale. Read out all options.

[1]	not at all likely
[2]	little likely
[3]	somewhat likely
[4]	rather likely
[5]	very likely
[98]	no response given

[chron_no_ppe] [select_one]

135) How likely do you think it is that you suffer from chronical disease from using pesticides without wearing all the recommended PPE?

Cheat sheet 5 - Use 5 dots scale. Read out all options.

[1]	not at all likely
[2]	little likely
[3]	somewhat likely
[4]	rather likely
[5]	very likely
[98]	no response given

[cons_own] [select_one]

136) Imagine you are suffering from acute symptoms of pesticide usage, how severe do you rate the consequences for your own health?

Cheat sheet 5 - Use 5 dots scale. Read out all options.

[1]	not at all severe
[2]	a little severe
[3]	somewhat severe
[4]	rather severe
[5]	very severe
[98]	no response given

[cons_fam] [select_one]

137) Imagine you are suffering from chronic disease due to pesticide usage, how severe do you rate the consequences for your family's (e.g. your childrens') life?

Cheat sheet 5 - Use 5 dots scale. Read out all options.

[1]	not at all severe
[2]	a little severe
[3]	somewhat severe
[4]	rather severe
[5]	very severe
[98]	no response given

[aff_fam] [select_one]

138) How strongly are pesticides currently affecting you or your family's health at the moment?

Cheat sheet 5 - Use 5 dots scale. Read out all options.

[1]	not at all
[2]	a little
[3]	somewhat/ to a medium extent
[4]	rather well / much
[5]	very much
[98]	no response given

• Relevant when:
5) Does the farmer agree to take part in the survey? was answered with yes.

cross_behavior

[ ] [note]
Listed below are some statements about the relationship between PPE use. For each one, please indicate to what extend you agree with it.

[ ] [note]
Do you agree with the following statements:

[ppe_use6] [select_one]

139) As long as I wear all the recommended PPE when handling pesticides, I don't need to handle pesticides carefully.

Cheat sheet 5 - Use 5 dots scale. Read out all options.

[1]	agree not at all
[2]	agree a little
[3]	somewhat agree
[4]	rather agree
[5]	strongly agree
[98]	no response given

[ppe_use10] [select_one]

140) Using all the recommended PPE allows me to handle more pesticides with no consequences.

Cheat sheet 5 - Use 5 dots scale. Read out all options.

[1]	agree not at all
[2]	agree a little
[3]	somewhat agree
[4]	rather agree
[5]	strongly agree
[98]	no response given

• Relevant when:
5) Does the farmer agree to take part in the survey? was answered with yes.

[ ] [note]
# Please save the questionnaire

• Relevant when:
5) Does the farmer agree to take part in the survey? was answered with yes.

[ ] [note]
# Thanks for providing us all the inforamtion about pesticide use. Now we could like to continue with questions about your medical history

[ ] [note]
#F. Medical History of the Participant

• Relevant when:
5) Does the farmer agree to take part in the survey? was answered with yes.

[time_f] [time]
Please take the time

• Relevant when:
5) Does the farmer agree to take part in the survey? was answered with yes.

F. Medical History of the Participant

[med_covid] [select_one]
141) Did suffer from COVID in PAST 12 MONTHS??

[0]	No
[1]	Yes, confirmed by test or doctor
[2]	Yes, experienced sympthoms but not diagnosed

• Relevant when:
141) Did suffer from COVID in PAST 12 MONTHS?? was answered with Yes, confirmed by test or doctor
OR 141) Did suffer from COVID in PAST 12 MONTHS?? was answered with Yes, experienced sympthoms but not diagnosed.

[med_covid_sym] [select_multiple]

141.1) if you suffer from COVID what were your symphoms you experienced?

let the particpant state the sympthoms and then slect them in the list, multiple selection possible

[1]	Fever
[2]	Cough
[3]	Tiredness
[4]	Loss of taste or smell
[5]	Sore throat
[6]	Headache
[7]	Aches and pains
[8]	Diarrhoea
[9]	A rash on skine, or discoloration of fingers or toes
[10]	Red or irritated eyes
[11]	Difficulty breathing or shortness of breath
[12]	Loss of speech or mobility, or confusion
[13]	Chest pain
[96]	Other

[99]	Don't know/none mentioned

• Relevant when:
141.1) if you suffer from COVID what were your symphoms you experienced? was answered with Other.

[med_covid_sym_sp] [text]
141.2) Specify other symptoms

[med_covid_vac] [select_one]
142) Are you vaccinated for COVID or do you have intentions to get vaccinated?

[1]	Yes, vaccinated
[2]	Not yet vaccianted but would like to if available
[3]	No intention to get vaccinated

- Relevant when:
142) Are you vaccinated for COVID or do you have intentions to get vaccinated? was answered with Yes, vaccinated.

[med_covid_vac2] [select_one]
142.1) Do you know if you are fully vaccinated?

[1]	I'm fully vaccinated

[2]	I have one vaccination and need a second one
[3]	I'm not sure

• Relevant when:
142) Are you vaccinated for COVID or do you have intentions to get vaccinated? was answered with No intention to get vaccinated.

[med_covid_vac_sp] [text]
142.1) Would you like to share your resons why you do not want to get vaccianted?

[med_12] [select_one]
143) Did suffer from any illnesses for more than five consecutive days in the PAST 12 MONTHS??
exept covid

[0]	No
[1]	Yes
[98]	Don't know

• Relevant when:
143) Did suffer from any illnesses for more than five consecutive days in the PAST 12 MONTHS?? was answered with Yes.

[med_13] [select_multiple]
143.1) Which illnesses did you suffer from in the PAST 12 MONTHS?
Don't read the options, select applicable illness groups

[1]	Physical problem / Pain / Accident / Injury
[2]	Infectious diseases
[3]	Chronic diseases
[96]	Other

• Relevant when:
143.1) Which illnesses did you suffer from in the PAST 12 MONTHS? was answered with Other.

[med_13_sp] [text]
143.2) Specify other illnesses in the PAST 12 MONTHS?

[med1] [select_multiple]

144) Have you taken any medications (oral, inhalation, intravenous or skin) for more than five consecutive days in the PAST 12 MONTHS?

Choose from list

[1]	Anti-fungal cream
[2]	Anti-Malaria
[3]	Antiallergic
[4]	Antibiotic
[5]	Antiretroviral theraphy
[6]	Flu / Cough
[7]	Herbal / Alternative medicine
[8]	Hypertension / Heart medicine
[9]	Injections
[10]	Painkiller
[11]	Skin cream
[12]	Ulcer medicine
[13]	Vitamins / Supplements
[96]	Other
[99]	None

• Relevant when:
5) Does the farmer agree to take part in the survey? was answered with yes.

[ ] [note]
# Please save the questionnaire

• Relevant when:
5) Does the farmer agree to take part in the survey? was answered with yes.

[ ] [note]
#G. Acute and Chronic Neurological Symptoms

• Relevant when:
5) Does the farmer agree to take part in the survey? was answered with yes.

G. Acute and Chronic Neurological Symptoms

[nq1] [note]

Read the following instructions to the participant:
"Now I am going to read you a list of symptoms. Please tell me if you have had these symptoms during the last 12 months.

Enter the answers directly on the tablet.
- If the answer to the question is "NO" continue with the next symptom.
-If the answer to a question is “YES”: Show the participant the template (Cheat sheet 4 - frequency, Awith the information about frequency and say "I would like to know how many times you felt this symptom. Look at this sheet; Is divided into tables with an increasing number of black dots: the table with the number 1 that has some points, represents "once a year"; The center table with the number 2 represents "once a month"; The box with the number 3 represents "once a week" and the last one with the number 4 that has more points, means "more than once a week”.

Enter this answer also on the tablet.
Say: Can you also tell me if you had this symptom after pesticide application during the past 12 month?
Continue with the next symptom.
Cheat sheet 8 - health frequency

[i01_u] [select_one]

Dizziness

Did you have dizziness during the last 12 months?

[0]	No
[1]	Yes
[98]	Don't know

• Relevant when:
Dizziness was answered with Yes.

145.i01 - Dizziness

[i01] [note]
145.i01 - Dizziness

[i01_v] [select_one]
b. How often you had this symptom during the last 12 months?

[1]	once a year
[2]	once a month
[3]	once a week
[4]	more than once a week
[98]	don't know

[i01_p] [select_one]

c. Did you have this symptom after pesticide application??

[0]	No
[1]	Yes
[98]	Don't know

[i02_u] [select_one]

Feeling tense, anxious or nervous

Did you feel tense, anxious or nervous during the last 12 months?

[0]	No
[1]	Yes
[98]	Don't know

• Relevant when:

Feeling tense, anxious or nervous was answered with Yes.

145.i02 - Feeling tense, anxious or nervous

[i02] [note]

145.i02 - Feeling tense, anxious or nervous

[i02_v] [select_one]

b. How often you had this symptom during the last 12 months?

[1]	once a year
[2]	once a month
[3]	once a week
[4]	more than once a week
[98]	don't know

[i02_p] [select_one]

c. Did you have this symptomafter pesticide application?

[0]	No
[1]	Yes
[98]	Don't know

[i03_u] [select_one]
Nausea
Did you suffer from nausea during the last 12 months?

[0]	No
[1]	Yes
[98]	Don't know

• Relevant when:
Nausea was answered with Yes.

145.i03 - Nausea

[i03] [note]
145.i03 - Nausea

[i03_v] [select_one]

b. How often you had this symptom during the last 12 months?

[1]	once a year
[2]	once a month
[3]	once a week
[4]	more than once a week
[98]	don't know

[i03_p] [select_one]

c. Did you have this symptom after pesticide application?

[0]	No
[1]	Yes
[98]	Don't know

[i04_u] [select_one]

Vomiting

[0]	No
[1]	Yes
[98]	Don't know

• Relevant when:
Vomiting was answered with Yes.

145.i04 - Vomiting

[i04] [note]
145.i04 - Vomiting

[i04_v] [select_one]
b. How often you had this symptom during the last 12 months?

[1]	once a year
[2]	once a month
[3]	once a week
[4]	more than once a week
[98]	don't know

[i04_p] [select_one]
c. Did you have this symptom after pesticide application?

[0]	No
[1]	Yes
[98]	Don't know

[i05_u] [select_one]
Diarrhea

[0]	No
[1]	Yes
[98]	Don't know

• Relevant when:
Diarrhea was answered with Yes.

145.i05 - Diarrhea

[i05] [note]
145.i05 - Diarrhea

[i05_v] [select_one]

b. How often you had this symptom during the last 12 months?

[1]	once a year
[2]	once a month
[3]	once a week
[4]	more than once a week
[98]	don't know

[i05_p] [select_one]

c. Did you have this symptom after pesticide application?

[0]	No
[1]	Yes
[98]	Don't know

[i06_u] [select_one]

Feeling tired, sleepy or with little energy most of the day

[0]	No
[1]	Yes
[98]	Don't know

• Relevant when:
Feeling tired, sleepy or with little energy most of the day was answered with Yes.

145.i06 - Feeling tired, sleepy or with little energy most of the day

[i06] [note]
145.i06 - Feeling tired, sleepy or with little energy most of the day

[i06_v] [select_one]
b. How often you had this symptom during the last 12 months?

[1]	once a year
[2]	once a month
[3]	once a week
[4]	more than once a week
[98]	don't know

[i06_p] [select_one]

c. Did you have this symptom after pesticide application?

[0]	No
[1]	Yes
[98]	Don't know

[i07_u] [select_one]

Sweating more than normal

[0]	No
[1]	Yes
[98]	Don't know

• Relevant when:

Sweating more than normal was answered with Yes.

145.i07 - Sweating more than normal

[i07] [note]

145.i07 - Sweating more than normal

[i07_v] [select_one]

b. How often you had this symptom during the last 12 months?

[1]	once a year
[2]	once a month
[3]	once a week
[4]	more than once a week
[98]	don't know

[i07_p] [select_one]

c. Did you have this symptom after pesticide application?

[0]	No
[1]	Yes
[98]	Don't know

[i08_u] [select_one]

Salivation more than normal

[0]	No
[1]	Yes
[98]	Don't know

• Relevant when:
Salivation more than normal was answered with Yes.

145.i08 - Salivation more than normal

[i08] [note]
145.i08 - Salivation more than normal

[i08_v] [select_one]
b. How often you had this symptom during the last 12 months?

[1]	once a year
[2]	once a month
[3]	once a week
[4]	more than once a week
[98]	don't know

[i08_p] [select_one]

c. Did you have this symptom after pesticide application?

[0]	No
[1]	Yes
[98]	Don't know

[i09_u] [select_one]

Difficulties to see during the night

[0]	No
[1]	Yes
[98]	Don't know

• Relevant when:

Difficulties to see during the night was answered with Yes.

145.i09 - Difficulties to see during the night

[i09] [note]

145.i09 - Difficulties to see during the night

[i09_v] [select_one]

b. How often you had this symptom during the last 12 months?

[1]	once a year
[2]	once a month
[3]	once a week
[4]	more than once a week
[98]	don't know

[i09_p] [select_one]

c. Did you have this symptom after pesticide application?

[0]	No
[1]	Yes
[98]	Don't know

[i10_u] [select_one]

Feeling distracted or confused

[0]	No
[1]	Yes
[98]	Don't know

• Relevant when:
Feeling distracted or confused was answered with Yes.

145.i10 - Feeling distracted or confused

[i10] [note]
145.i10 - Feeling distracted or confused

[i10_v] [select_one]
b. How often you had this symptom during the last 12 months?

[1]	once a year
[2]	once a month
[3]	once a week
[4]	more than once a week
[98]	don't know

[i10_p] [select_one]
c. Did you have this symptom after pesticide application?

[0]	No
[1]	Yes
[98]	Don't know

[i11_u] [select_one]
Paleness

[0]	No
[1]	Yes
[98]	Don't know

• Relevant when:
Paleness was answered with Yes.

145.i11 - Paleness

[i11] [note]
145.i11 - Paleness

[i11_v] [select_one]

b. How often you had this symptom during the last 12 months?

[1]	once a year
[2]	once a month
[3]	once a week
[4]	more than once a week
[98]	don't know

[i11_p] [select_one]

c. Did you have this symptom after pesticide application?

[0]	No
[1]	Yes
[98]	Don't know

[i12_u] [select_one]

Headache

[0]	No
[1]	Yes
[98]	Don't know

• Relevant when:
Headache was answered with Yes.

145.i12 - Headache

[i12] [note]
145.i12 - Headache

[i12_v] [select_one]
b. How often you had this symptom during the last 12 months?

[1]	once a year
[2]	once a month
[3]	once a week
[4]	more than once a week
[98]	don't know

[i12_p] [select_one]
c. Did you have this symptom after pesticide application?

[0]	No
[1]	Yes
[98]	Don't know

[i13_u] [select_one]
Earache

[0]	No
[1]	Yes
[98]	Don't know

• Relevant when:
Earache was answered with Yes.

145.i13 - Earache

[i13] [note]
145.i13 - Earache

[i13_v] [select_one]

b. How often you had this symptom during the last 12 months?

[1]	once a year
[2]	once a month
[3]	once a week
[4]	more than once a week
[98]	don't know

[i13_p] [select_one]

c. Did you have this symptom after pesticide application?

[0]	No
[1]	Yes
[98]	Don't know

[i14_u] [select_one]

Changes in appetite

[0]	No
[1]	Yes
[98]	Don't know

• Relevant when:
Changes in appetite was answered with Yes.

145.i14 - Changes in appetite

[i14] [note]
145.i14 - Changes in appetite

[i14_v] [select_one]
b. How often you had this symptom during the last 12 months?

[1]	once a year
[2]	once a month
[3]	once a week
[4]	more than once a week
[98]	don't know

[i14_p] [select_one]

c. Did you have this symptom after pesticide application?

[0]	No
[1]	Yes
[98]	Don't know

[i15_u] [select_one]

Fast heart rate

[0]	No
[1]	Yes
[98]	Don't know

• Relevant when:

Fast heart rate was answered with Yes.

145.i15 - Fast heart rate

[i15] [note]

145.i15 - Fast heart rate

[i15_v] [select_one]

b. How often you had this symptom during the last 12 months?

[1]	once a year
[2]	once a month
[3]	once a week
[4]	more than once a week
[98]	don't know

[i15_p] [select_one]

c. Did you have this symptom after pesticide application?

[0]	No
[1]	Yes
[98]	Don't know

[i16_u] [select_one]

Problems or difficulties to maintain balance

[0]	No
[1]	Yes
[98]	Don't know

• Relevant when:
Problems or difficulties to maintain balance was answered with Yes.

145.i16 - Problems or difficulties to maintain balance

[i16] [note]
145.i16 - Problems or difficulties to maintain balance

[i16_v] [select_one]
b. How often you had this symptom during the last 12 months?

[1]	once a year
[2]	once a month
[3]	once a week
[4]	more than once a week
[98]	don't know

[i16_p] [select_one]
c. Did you have this symptom after pesticide application?

[0]	No
[1]	Yes
[98]	Don't know

[i17_u] [select_one]
Blurred or double vision

[0]	No
[1]	Yes
[98]	Don't know

• Relevant when:
Blurred or double vision was answered with Yes.

145.i17 - Blurred or double vision

[i17] [note]
145.i17 - Blurred or double vision

[i17_v] [select_one]

b. How often you had this symptom during the last 12 months?

[1]	once a year
[2]	once a month
[3]	once a week
[4]	more than once a week
[98]	don't know

[i17_p] [select_one]

c. Did you have this symptom after pesticide application?

[0]	No
[1]	Yes
[98]	Don't know

[i18_u] [select_one]

Problems or difficulties with concentration

[0]	No
[1]	Yes
[98]	Don't know

• Relevant when:
Problems or difficulties with concentration was answered with Yes.

145.i18 - Problems or difficulties with concentration

[i18] [note]
145.i18 - Problems or difficulties with concentration

[i18_v] [select_one]
b. How often you had this symptom during the last 12 months?

[1]	once a year
[2]	once a month
[3]	once a week
[4]	more than once a week
[98]	don't know

[i18_p] [select_one]

c. Did you have this symptom after pesticide application?

[0]	No
[1]	Yes
[98]	Don't know

[i19_u] [select_one]

Tingling, numbness or feeling of pins and needles in hands or feet

[0]	No
[1]	Yes
[98]	Don't know

• Relevant when:

Tingling, numbness or feeling of pins and needles in hands or feet was answered with Yes.

145.i19 - Tingling, numbness or feeling of pins and needles in hands or feet

[i19] [note]

145.i19 - Tingling, numbness or feeling of pins and needles in hands or feet

[i19_v] [select_one]

b. How often you had this symptom during the last 12 months?

[1]	once a year
[2]	once a month
[3]	once a week
[4]	more than once a week
[98]	don't know

[i19_p] [select_one]

c. Did you have this symptom after pesticide application?

[0]	No
[1]	Yes
[98]	Don't know

[i20_u] [select_one]

Feeling fearful

[0]	No
[1]	Yes
[98]	Don't know

• Relevant when:
Feeling fearful was answered with Yes.

145.i20 - Feeling fearful

[i20] [note]
145.i20 - Feeling fearful

[i20_v] [select_one]
b. How often you had this symptom during the last 12 months?

[1]	once a year
[2]	once a month
[3]	once a week
[4]	more than once a week
[98]	don't know

[i20_p] [select_one]

c. Did you have this symptom after pesticide application?

[0]	No
[1]	Yes
[98]	Don't know

[i21_u] [select_one]

Loss of consciousness or fainting

[0]	No
[1]	Yes
[98]	Don't know

• Relevant when:
Loss of consciousness or fainting was answered with Yes.

145.i21 - Loss of consciousness or fainting

[i21] [note]

145.i21 - Loss of consciousness or fainting

[i21_v] [select_one]

b. How often you had this symptom during the last 12 months?

[1]	once a year
[2]	once a month
[3]	once a week
[4]	more than once a week
[98]	don't know

[i21_p] [select_one]

c. Did you have this symptom after pesticide application?

[0]	No
[1]	Yes
[98]	Don't know

[i22_u] [select_one]

Feeling irritable or angry more than normal

[0]	No
[1]	Yes
[98]	Don't know

• Relevant when:
Feeling irritable or angry more than normal was answered with Yes.

145.i22 - Feeling irritable or angry more than normal

[i22] [note]
145.i22 - Feeling irritable or angry more than normal

[i22_v] [select_one]
b. How often you had this symptom during the last 12 months?

[1]	once a year
[2]	once a month
[3]	once a week
[4]	more than once a week
[98]	don't know

[i22_p] [select_one]
c. Did you have this symptom after pesticide application?

[0]	No
[1]	Yes
[98]	Don't know

[i23_u] [select_one]
Stomach or abdominal pain

[0]	No
[1]	Yes
[98]	Don't know

• Relevant when:
Stomach or abdominal pain was answered with Yes.

145.i23 - Stomach or abdominal pain

[i23] [note]
145.i23 - Stomach or abdominal pain

[i23_v] [select_one]

b. How often you had this symptom during the last 12 months?

[1]	once a year
[2]	once a month
[3]	once a week
[4]	more than once a week
[98]	don't know

[i23_p] [select_one]

c. Did you have this symptom after pesticide application?

[0]	No
[1]	Yes
[98]	Don't know

[i24_u] [select_one]

Tremor / Shaking hands

[0]	No
[1]	Yes
[98]	Don't know

• Relevant when:
Tremor / Shaking hands was answered with Yes.

145.i24 - Tremor / Shaking hands

[i24] [note]
145.i24 - Tremor / Shaking hands

[i24_v] [select_one]
b. How often you had this symptom during the last 12 months?

[1]	once a year
[2]	once a month
[3]	once a week
[4]	more than once a week
[98]	don't know

[i24_p] [select_one]
c. Did you have this symptom after pesticide application?

[0]	No
[1]	Yes
[98]	Don't know

[i25_u] [select_one]
Problems or difficulties with sleeping or falling asleep

[0]	No
[1]	Yes
[98]	Don't know

• Relevant when:
Problems or difficulties with sleeping or falling asleep was answered with Yes.

145.i25 - Problems or difficulties with sleeping or falling asleep

[i25] [note]
145.i25 - Problems or difficulties with sleeping or falling asleep

[i25_v] [select_one]
b. How often you had this symptom during the last 12 months?

[1]	once a year
[2]	once a month
[3]	once a week
[4]	more than once a week
[98]	don't know

[i25_p] [select_one]
c. Did you have this symptom after pesticide application?

[0]	No
[1]	Yes
[98]	Don't know

[i26_u] [select_one]
Problems or difficulties with talking

[0]	No
[1]	Yes
[98]	Don't know

• Relevant when:
Problems or difficulties with talking was answered with Yes.

145.i26 - Problems or difficulties with talking

[i26] [note]
145.i26 - Problems or difficulties with talking

[i26_v] [select_one]
b. How often you have this symptom during the last 12 months?

[1]	once a year
[2]	once a month
[3]	once a week
[4]	more than once a week
[98]	don't know

[i26_p] [select_one]
c. Did you have this symptom after pesticide application?

[0]	No
[1]	Yes
[98]	Don't know

[i27_u] [select_one]
Weakness of arms or legs

[0]	No
[1]	Yes
[98]	Don't know

• Relevant when:
Weakness of arms or legs was answered with Yes.

145.i27 - Weakness of arms or legs

[i27] [note]
145.i27 - Weakness of arms or legs

[i27_v] [select_one]

b. How often you had this symptom during the last 12 months?

[1]	once a year
[2]	once a month
[3]	once a week
[4]	more than once a week
[98]	don't know

[i27_p] [select_one]

c. Did you have this symptom after pesticide application?

[0]	No
[1]	Yes
[98]	Don't know

[i28_u] [select_one]

Changes in taste or smell

[0]	No
[1]	Yes
[98]	Don't know

• Relevant when:
Changes in taste or smell was answered with Yes.

145.i28 - Changes in taste or smell

[i28] [note]
145.i28 - Changes in taste or smell

[i28_v] [select_one]
b. How often you had this symptom during the last 12 months?

[1]	once a year
[2]	once a month
[3]	once a week
[4]	more than once a week
[98]	don't know

[i28_p] [select_one]
c. Did you have this symptom after pesticide application?

[0]	No
[1]	Yes
[98]	Don't know

[i29_u] [select_one]
Unsecure walk or afraid to fall

[0]	No
[1]	Yes
[98]	Don't know

• Relevant when:
Unsecure walk or afraid to fall was answered with Yes.

145.i29 - Unsecure walk or afraid to fall

[i29] [note]
145.i29 - Unsecure walk or afraid to fall

[i29_v] [select_one]

b. How often you had this symptom during the last 12 months?

[1]	once a year
[2]	once a month
[3]	once a week
[4]	more than once a week
[98]	don't know

[i29_p] [select_one]

c. Did you have this symptom after pesticide application?

[0]	No
[1]	Yes
[98]	Don't know

[i30_u] [select_one]

Feeling depressed, apathetic or tired

[0]	No
[1]	Yes
[98]	Don't know

• Relevant when:
Feeling depressed, apathetic or tired was answered with Yes.

145.i30 - Feeling depressed, apathetic or tired

[i30] [note]
145.i30 - Feeling depressed, apathetic or tired

[i30_v] [select_one]
b. How often you had this symptom during the last 12 months?

[1]	once a year
[2]	once a month
[3]	once a week
[4]	more than once a week
[98]	don't know

[i30_p] [select_one]
c. Did you have this symptom after pesticide application?

[0]	No
[1]	Yes
[98]	Don't know

[i31_u] [select_one]
Jerking or involuntary movements in arms or legs

[0]	No
[1]	Yes
[98]	Don't know

• Relevant when:
Jerking or involuntary movements in arms or legs was answered with Yes.

145.i31 - Jerking or involuntary movements in arms or legs

[i31] [note]
145.i31 - Jerking or involuntary movements in arms or legs

[i31_v] [select_one]
b. How often you had this symptom during the last 12 months?

[1]	once a year
[2]	once a month
[3]	once a week
[4]	more than once a week
[98]	don't know

[i31_p] [select_one]
c. Did you have this symptom after pesticide application?

[0]	No
[1]	Yes
[98]	Don't know

[ ] [note]
#H. Pesticide Intoxication

• Relevant when:

5) Does the farmer agree to take part in the survey? was answered with yes.

H. Pesticide Intoxication

[i32] [text]

146) Did you experience any other symptom after pesticide application which we did not ask for? If yes, which ones?

only symphoms that have not been mentioned before

[int_cu] [integer]

147) How many times have you experienced any of the symptoms you just mentioned after applying a pesticid last year?

remind the farmer about the past 12 month o f the cheat sheet 1

[int_do] [integer]

148) How many times have you visited a doctor and a pesticide poisioning was diagnosed last year?

times, write 0 if no sympthoms were mentioned before

[int_life] [select_one]

149) Apart from last year, have you ever in your life experienced any of the sypmtoms you just mentioned after applying a pesticid ?

times, excluding last year

[0]	No
[1]	Yes
[98]	Don't know

[int_life_do] [integer]

150) How many times have you visited a doctor and a pesticide poisioning was diagnosed?

times, excluding last year, write 0 if no sympthoms were mentiond

• Relevant when:

5) Does the farmer agree to take part in the survey? was answered with yes.

[time_final] [time]

take the current time

• Relevant when:
(7) To which intervention group did the participant belong? was answered with 2-day training + SMS) OR (7) To which intervention group did the participant belong? was answered with 2-day training).

[int_2day] [select_one]
151) How much did you like the 2-day training?
use cheat sheet 5

[1]	not at all
[2]	a little
[3]	somewhat/ to a medium extent
[4]	rather well / much
[5]	very much
[98]	no response given

• Relevant when:

(7) To which intervention group did the participant belong? was answered with 2-day training + SMS) OR
(7) To which intervention group did the participant belong? was answered with 2-day training).

[int_2day_farm] [select_one]

152) How effective did you find 2-day training to help you improve your pesticde related safety and health?

use cheat sheet 6

[1]	not at all
[2]	a little
[3]	somewhat/ to a medium extent
[4]	rather well / much
[5]	very much
[98]	no response given

• Relevant when:

(7) To which intervention group did the participant belong? was answered with 2-day training + SMS) OR
(7) To which intervention group did the participant belong? was answered with 2-day training).

[int_2day_like] [text]

153) Which part of the 2-day training did you like most

• Relevant when:
(7) To which intervention group did the participant belong? was answered with 2-day training + SMS) OR (7) To which intervention group did the participant belong? was answered with 2-day training).

[int_2day_improve] [text]

154) What would you improve on the 2-day training?

• Relevant when:
7) To which intervention group did the participant belong? was answered with 2-day training + SMS.

[int_sms] [select_one]

155) How much did you like the SMS survey?

use cheat sheet 5

[1]	not at all
[2]	a little
[3]	somewhat/ to a medium extent
[4]	rather well / much
[5]	very much
[98]	no response given

• Relevant when:
7) To which intervention group did the participant belong? was answered with 2-day training + SMS.

[int_sms_farm] [select_one]

156) How effective did you find SMS to help you improve your pesticde related safety and health?

use cheat sheet 6

[1]	not at all
[2]	a little
[3]	somewhat/ to a medium extent
[4]	rather well / much
[5]	very much
[98]	no response given

• Relevant when:
7) To which intervention group did the participant belong? was answered with 2-day training + SMS.

[int_sms_like] [text]

157) Which part of the SMS intervention did you like most

• Relevant when:
7) To which intervention group did the participant belong? was answered with 2-day training + SMS.

[int_sms_improve] [text]
158) What would you improve on the SMS Intervention?

• Relevant when:
5) Does the farmer agree to take part in the survey? was answered with yes.

[sta_9] [note]
The interview portion of this visit is complete. Thanks for your attention.

• Relevant when:
5) Does the farmer agree to take part in the survey? was answered with yes.

[clas] [select_one]
How would you rate the informations provided by the participant?
if bad please metion the reasons also to your supervisor

[1]	Good
[2]	Regular
[3]	Bad

• Relevant when:
5) Does the farmer agree to take part in the survey? was answered with yes.

[obs] [text]
Observations:
if too long to record make an note in your booklet and discuss the reporting with your supervisor

• Relevant when:
5) Does the farmer agree to take part in the survey? was answered with yes.

[ ] [note]
Make sure to stop the voice recording and save as id-q4-date
