# Supplementary material for: Evaluating and Enhancing an Educational Intervention to Reduce Smallholder Farmers’ Exposure to Pesticides in Uganda Through a Digital, Systematic Approach to Behavior Change: Protocol for a Cluster-Randomized Controlled Trial
Source: JMIR Res Protoc. 2024 May 8;13:e55238. doi: 10.2196/55238 (PMC11112482; doi:10.2196/55238)
Supplement: Multimedia Appendix 6 [file resprot_v13i1e55238_app6.docx]

**Knowledge, attitude and practice questions along the four modules of the education curriculum on safe pesticide handling**

Table 2: Knowledge, attitude and practice questions along the four modules of the education curriculum on safe pesticide handling

| **Description of sections** |  | **KNOWLEDGE** | | **ATTITUDES** | | | **PRACTICES** | | |
| --- | --- | --- | --- | --- | --- | --- | --- | --- | --- |
| **Instructions** | Positive (p)/  negative (n) | Variable name | Question In your opinion how many % of the smallholder farmers you are working with would think this statement is true?  95 Do not have an opinion | Positive (p)/  negative (n) | variable name | Question In your opinion how strongly would the average smallholder farmer you are working with agree with following statements: 1 agree not at all 2 agree a little 3 somewhat agree 4 rather agree 5 strongly agree  95 Do not have an opinion | Positive (p)/  negative (n) | variable name | Question In your opinion how often would an average smallholder farmer you are working with apply following practice? 1 Never (0%) 2 Rarely (25%) 3 Sometimes (50%) 4 Often (75%) 5 Always (100%)  95 Do not have an opinion |
| **1. General safety statements about Plant protection product (PPP) handling** | **p** | **k1** | 50) The name of the PPP active ingredients can be found on the label of the product | **p** | **a1** | 67) It is important for me to know the active ingredients in a given PPP | **P** | **p1** | 83) I look for the name of the active ingredients when buying or using a given PPP |
|  | **p** | **k2** | 51) The PPP label contains relevant information on how to handle and use the product | **p** | **a2** | 68) It is necessary to read instructions on PPP label | **P** | **p2** | 84) When buying and before using a new PPP I read (or ask someone to read for me) the instructions on the label |
|  | **n** | **k3** | 52) The dose recommended on the label is only a suggestion and should be adapted by the farmer according to needs. | **n** | **a3** | 70) If you follow the mixing rate on the label, the PPP doesn't not work well | **N** | **p3** | 85) I mix stronger doses of the PPPs that I use on my crops to make sure it works |
|  | **p** | **k4** | 53) When one PPP is used frequently, pests get used to it and develop resistance | **n** | **a4** | 71) Pests only develop resistance to a PPP if it is fake/counterfeit | **P** | **p4** | 86) I change/alternate the PPPs I use based on different modes of action |
| **2. PPP and human health** | **p** | **k5** | 54) Empty PPP containers should never be reused for packing any food stuff at home | **n** | **a5** | 72) When washed well, an empty PPP container is safe to use at home | **N** | **p5** | 87) In my home, we reuse empty PPP containers for packing things such as sugar, salt, paraffin and others |
|  | **n** | **k6** | 55) By smelling on a PPP, one can tell how toxic/hazardous it is | **n** | **a6** | 73) As a farmer I am not interested in knowing the hazard level of a PPP as long as it kills the pests on my farm | **P** | **p6** | 88) I look at the colour codes on a PPP label to tell the hazard level of that PPP |
|  | **p** | **k7** | 56) PPPs have no effects on the health of children | **n** | **a7** | 74) Involving children in mixing and applying PPPs  helps to equip them with farming skills at a young age | **P** | **p7** | 89) In my home, I don’t allow my children to handle PPPs |
|  | **n** | **k8** | 57) One can only get exposed to PPPs during spraying | **n** | **a8** | 75) I am comfortable working in the field on the same day that I spray it | **N** | **p8** | 90) I do some other tasks in my field immediately or a few hours after spraying it with PPPs |
| **3. PPPs and the environment** | **p** | **k9** | 58) PPPs travel in space and pollute the air we breathe in | **n** | **a9** | 76) When spraying, PPP droplets are too tiny to drift and contaminate a nearby water point | **P** | **p9** | 91) I only spray when it’s not windy to avoid PPPs drifting into the air |
|  | **p** | **k10** | 59) Leaving a distance of at least 5 meters between your garden and a nearby water way is a responsible practice | **n** | **a10** | Planting as close as possible to waterbody is a good practice to maximize the land | **P** | **p10** | 92) I apply PPPs within less than 5m to nearby water ways/sources |
|  | **n** | **k11** | 60) PPPs which kill all the insects in the field are the most effective | **n** | **a11** | 77) If there are many pests in the field then one should make the spraying mixture stronger | **n** | **p11** | 93) I mix strong concentrations so that I can kill all the insects in my field |
|  | **n** | **k12** | 61) PPP containers can be discharged with the normal waste | **n** | **a12** | 78) It is not of my concern how the empty PPP containers are discharged | **n** | **p12** | 94) I leave my empty PPP containers in the field or burn them |
| **4. PPP application** | **p** | **k13** | 62) Checking your spraying equipment for any leakages before mixing is a good practice | **p** | **a13** | 79) Testing one’s knapsack sprayer with water before mixing PPPs can save him/her from getting exposed while spraying | **p** | **p13** | 95) I test my spraying equipment with water before the PPP application |
|  | **n** | **k14** | 63) The best time to spray is during hot/sunny times of the day | **n** | **a14** | 80) Personal protective clothing should only be worn when it is not too hot | **n** | **p14** | 96) I mix and spray PPPs in my field independent of the weather condition |
|  | **n** | **k15** | 64) It is a good practice to have one spraying equipment which can do all kinds of spraying including animals and crops | **n** | **a15** | 81) Having separate spraying equipment for crops and animals is a wastage of money | **n** | **P15** | 97) I use the same spraying equipment for my crops and animals |
|  | **p** | **k16** | 65) A good sprayer should have different types of nozzles | **n** | **a16** | 82) It is unnecessary to have more than one nozzle type for the same spraying equipment | **p** | **p16** | 98) With my spraying equipment, I use different nozzles, depending on what I am spraying against |
